# A novel mouse model of upper tract urothelial carcinoma highlights the impact of dietary intervention on gut microbiota and carcinogenesis prevention despite carcinogen exposure

**DOI:** 10.1002/ijc.35295

**Published:** 2024-12-18

**Authors:** Akinaru Yamamoto, Atsunari Kawashima, Toshihiro Uemura, Kosuke Nakano, Makoto Matsushita, Yu Ishizuya, Kentaro Jingushi, Hiroaki Hase, Kotoe Katayama, Rui Yamaguchi, Nesrine Sassi, Yuichi Motoyama, Satoshi Nojima, Masashi Mita, Tomonori Kimura, Daisuke Motooka, Yuki Horibe, Yohei Okuda, Toshiki Oka, Gaku Yamamichi, Eisuke Tomiyama, Yoko Koh, Yoshiyuki Yamamoto, Taigo Kato, Koji Hatano, Motohide Uemura, Seiya Imoto, Hisashi Wada, Eiichi Morii, Kazutake Tsujikawa, Norio Nonomura

**Affiliations:** ^1^ Department of Urology, Graduate School of Medicine Osaka University Suita Osaka Japan; ^2^ Laboratory of Molecular and Cellular Physiology, Graduate School of Pharmaceutical Sciences Osaka University Suita Osaka Japan; ^3^ Laboratory of Sequence Analysis, Human Genome Center, The Institute of Medical Science The University of Tokyo Tokyo Japan; ^4^ Division of Cancer Systems Biology Aichi Cancer Center Research Institute Nagoya Japan; ^5^ Division of Cancer Informatics Nagoya University Graduate School of Medicine Nagoya Japan; ^6^ Department of Pathology, Graduate School of Medicine Osaka University Suita Osaka Japan; ^7^ KAGAMI Inc. Ibaraki Osaka Japan; ^8^ Department of Nephrology, Graduate School of Medicine Osaka University Suita Osaka Japan; ^9^ Department of Infection Metagenomics, Research Institute for Microbial Diseases Osaka University Suita Osaka Japan; ^10^ Division of Health Medical Intelligence, Human Genome Center, The Institute of Medical Science The University of Tokyo Tokyo Japan; ^11^ Department of Clinical Research in Tumor Immunology, Graduate School of Medicine Osaka University Suita Osaka Japan

**Keywords:** animal model, cancer prevention, microbiome, *N*‐butyl‐*N*‐(4‐hydroxybutyl) nitrosamine, upper tract urothelial carcinoma

## Abstract

Animal models of *N*‐butyl‐*N*‐(4‐hydroxy butyl) nitrosamine (BBN)‐induced urothelial carcinoma (UC), particularly bladder cancer (BC), have long been established. However, the rare incidence of BBN‐induced upper urinary tract UC (UTUC), which originates from the same urothelium as BC, remains elusive. The scarcity of animal models of UTUC has made it challenging to study the biology of UTUC. To address this problem, we tried to establish a novel mouse model of UTUC by treating multiple mice strains and sexes with BBN. The molecular consistency between the UTUC mouse model and human UTUC was confirmed using multi‐omics analyses, including whole‐exome, whole‐transcriptome, and spatial transcriptome sequencing. 16S ribosomal RNA metagenome sequencing, metabolome analysis, and dietary interventions were employed to assess changes in the gut microbiome, metabolome, and carcinogenesis of UTUC. Of all treated mice, only female BALB/c mice developed UTUC over BC. Multi‐omics analyses confirmed that the UTUC model reflected the molecular characteristics and heterogeneity of human UTUC with poor prognosis. Furthermore, the model exhibited increased *Tnf*‐related inflammatory gene expression in the upper urinary tract and a low relative abundance of *Parabacteroides distasonis* in the gut. Dietary intervention, mainly without alanine, led to *P. distasonis* upregulation and successfully prevented UTUC, as well as suppressed *Tnf*‐related inflammatory gene expression in the upper urinary tract despite the exposure to BBN. This is the first report to demonstrate a higher incidence of UTUC than BC in a non‐engineered mouse model using BBN. Overall, this model could serve as a useful tool for comprehensively investigating UTUC in future studies.

## Graphical abstract

What's new?

The BBN‐driven bladder cancer (BC) mouse model has been commonly used for more than 50 years. However, the mechanisms underlying the selective induction of BC over upper urinary tract urothelial carcinoma (UTUC) remain unclear. Here, the authors established a non‐genetically modified mouse strain with a higher incidence of UTUC following BBN administration. The study revealed nuanced metabolic and microbiome differences in the gut between UTUC and bladder cancer. Moreover, dietary intervention upregulated P. *distasonis* and prevented UTUC, also suppressing upper urinary tract inflammation despite BBN exposure. The new model may provide a useful tool for comprehensively investigating UTUC.

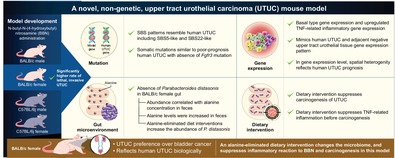

## 1 INTRODUCTION

Urothelial carcinoma (UC), which includes bladder cancer (BC) and upper urinary tract UC (UTUC), ranked fourth in incidence among men and twelfth among women in the United States in 2022.^1^ UTUC accounts for only approximately 5%–10% of all UCs, positioning it as a rare disease.^2^ Although BC and UTUC originate from the same urothelium lining the urinary tract^3^ and are generally managed as similar diseases in clinical practice, they exhibit different germplasm origins,^4^ patterns of occurrence,^5^, ^6^ molecular profiles,^7^, ^8^ and the clinical efficacy of systemic therapies.^9^, ^10^ To date, the differences in the mechanisms of UTUC and BC have been difficult to examine because of the scarcity of a UTUC animal model.

The *N*‐butyl‐*N*‐(4‐hydroxybutyl) nitrosamine (BBN)‐driven BC mouse model^11^ using C57BL/6 male mice has been commonly used for more than 50 years.^11^ However, studies on UTUC carcinogenesis are notably limited, and the mechanisms underlying the selective induction of BC over UTUC following BBN exposure in mice remain unclear. There are only a few reports on non‐engineered UTUC carcinogenesis mouse models using BBN^12^, ^13^; however, the carcinogenesis rate of UTUC was observed to be lower than that of BC. Saito reported that conditional double deletion of Trp53 and Pten in Upk3a‐positive cells caused UTUC in approximately one‐third of the mice; however, this was lower incidence than that of BC.^14^

In this study, we identified a novel non‐genetically modified mouse strain with a high incidence of UTUC over BC carcinogenesis following BBN administration. We explored the molecular concordance between this novel BBN‐induced UTUC mouse model and human UTUC using whole‐exome sequencing (WES), whole‐transcriptome sequencing (WTS), and spatial transcriptome sequencing. Additionally, we examined the effects of the gut microbiome and metabolome on BBN‐induced UTUC carcinogenesis using 16S ribosomal RNA metagenome sequencing (16S rRNA‐seq) and metabolome analysis. Finally, we assessed the role of dietary intervention‐induced changes in gut microbiota in BBN‐induced UTUC carcinogenesis prevention.

## 2 METHODS

### 2.1 Animals

C57BL/6j and BALB/c mice were purchased from Japan SLC (Tokyo, Japan) and maintained on a standard diet (MF; Oriental Yeast, Tokyo, Japan). Animals were sacrificed ethically using isoflurane inhalation anesthesia when significant weight loss, stooped posture, or gross hematuria were observed. Starting at 6–7 weeks of age, all experimental mice received 0.05% BBN (Tokyo Chemical Industry, Tokyo, Japan) until they were sacrificed, whereas control BALB/c female mice were given tap water. The animals were euthanized at 17–20 weeks after BBN administration. Samples were collected from the cancerous ureter (UT), and noncancerous ureter (UN) tissues of BBN‐treated mice, as well as, the healthy ureter (U) tissues of control mice. All samples were immediately preserved in RNAlater (Thermo Fisher Scientific, Waltham, MA, USA) and stored at −20°C. Fecal, and spleen samples were immediately frozen at −80°C after collection. Blood samples were collected from dissected mice and stored at 4°C overnight. Subsequently, serum was separated via centrifugation at 2000 × *g* for 15 min at 4°C, and the samples were stored at −80°C until further analysis.

### 2.2 Dietary intervention experiments

Mice in the dietary intervention group were fed a diet formulated according to the “AIN‐93G modified, no alanine added” diet (Oriental Yeast) (Supplementary Table S1) starting at 5 weeks of age. They were then administered 0.05% BBN starting at 6 weeks of age until the time of sacrifice. Mice in the normal diet group were fed an MF diet and similarly administered 0.05% BBN from 6 weeks of age until they were sacrificed. The final point of sacrifice was set at 17 weeks post‐BBN administration because sacrificial death was deemed necessary at that point.

### 2.3 Histopathological examinations

Histopathological examination was performed using hematoxylin and eosin (H&E) staining. Samples collected from mice were promptly infiltrated in 4% paraformaldehyde and stored at 4°C. The samples were embedded in paraffin, sectioned at a thickness of 4 μm, dehydrated with alcohol, and affixed to glass slides. For Ki‐67 and phospho‐histone H2A.X (γH2A.X) staining, formalin‐fixed and paraffin‐embedded (FFPE) tissue sections were utilized, followed by counterstaining with hematoxylin. Imaging was performed using a BZ‐X710 microscope (Keyence, Osaka, Japan). H&E, Ki‐67, and γH2A.X staining and diagnoses were performed by two histopathologists trained in light microscopy (S.N. and Y.M.). Positive Ki‐67 and γH2ax cells were counted in five different, randomly selected cancerous regions at a 400× magnification to calculate an average count. The following primary antibodies were used: rabbit anti‐mouse Ki‐67 (clone: D3B5; Cell Signaling Technology, Danvers, MA, USA) and rabbit anti‐mouse γH2A.X (Ser139) (clone: 20E3; Cell Signaling Technology).

For Ki‐67 and γH2A.X staining, 4‐μm sections of FFPE tissue were deparaffinized and subjected to antigen retrieval using a pressure cooker in citrate buffer (pH 7.0). Endogenous peroxidase activity was quenched using Dako REAL peroxidase‐blocking solution (Agilent Technologies, Santa Clara, CA, USA). Sections were incubated with primary antibodies diluted in Dako Antibody Diluent (Agilent Technologies) overnight at 4°C. For staining and detection, the Dako EnVision+ System‐ HRP‐Labeled Polymer Anti‐rabbit (Agilent Technologies) and Dako Liquid DAB+ Substrate Chromogen System (Agilent Technologies) were used in accordance with the manufacturer's instructions.

### 2.4 Reverse transcription‐quantitative PCR

RNA was extracted from each sample preserved in RNAlater at −20°C using the ISOSPIN Cell and Tissue RNA kit (Nippon Gene, Tokyo, Japan), following the manufacturer's standard protocol. The Prime Script RT Reagent Kit (Perfect Real Time) (Takara Bio, Shiga, Japan) was used to generate cDNA. Quantitative PCR (qPCR) was conducted on a CFX Connect Real‐Time System (Bio‐Rad, Hercules, CA, USA) using the THUNDERBIRD Next SYBR qPCR Mix (Toyobo, Osaka, Japan). The PCR protocol involved an initial denaturation at 95°C for 5 s, followed by 40 cycles of annealing and extension at 60°C for 30 s. Gene expression levels were quantified relative to that of the housekeeping gene, *Actb*, using the delta Ct method. Primer sequences were obtained from PrimerBank (https://pga.mgh.harvard.edu/primerbank/), and the specific primers used for qPCR are listed in Supplementary Table S2.

### 2.5 WES of the mouse model

DNA was extracted from samples preserved at −20°C in RNAlater using a chloroform‐based method. The library was prepared using the Twist EF 2.0 + Twist Mouse Exome Panel combination (Twist Bioscience, San Francisco, CA, USA). The extracted DNA was sequenced using the DNBSEQ‐G400RS platform (seq mode: 100 bp × 100 bp) to acquire genomic DNA. Genomic DNA was annotated and aligned against the GRCm38/mm10 reference genome using the Genomon pipeline (https://github.com/Genomon-Project/genomon-docs/tree/v2.0). Mutations were identified in the spleen tissue of the same individual as that of the control. Mutational signatures were analyzed using the R package, Mutationalpatterns.R^15^ Deconvolution was performed using AI through 1000 calculations. Similar single base substitution (SBS) patterns were defined with cutoff values of 0.71 for UN and 0.75 for UT. For the gene mutation list, we extracted genes common to more than eight patients (more than 10%) of the OU cohort and identified 18 genes. In addition, 47 mutated genes common to more than 10% of the patients were identified from previous reports in GeneBiology^16^ which are accessible from public databases. Mutation plots were generated using the R package, GenVisR for data visualization.^17^ The sequencing coverage and quality statistics for each sample are summarized in Supplementary Table S3.

### 2.6 WTS of the mouse model

RNA was extracted from each sample stored at −20°C in RNAlater using the miRNeasy Mini Kit (QIAGEN, Venlo, the Netherlands). The library was prepared using a combination of SMARTerHT (Takara Bio) and Nextera XT (Illumina, San Diego, CA, USA) kits, following the TAKARA protocol. The extracted RNA was sequenced on a DNBSEQ‐G400RS (seq mode: 100 bp × 100 bp) platform to acquire mRNA expression data. Gene expression levels were quantified by mapping the annotations to the GRCm38/mm10 reference genome. Gene expression was analyzed and evaluated using the free‐access platform, iDEP.^18^ An absolute fold change (FC) cutoff value of 2.0 and false discovery rate (FDR) <0.1 were used to identify differentially expressed genes (DEGs). After mapping these DEGs to the gene expression data of human samples, mouse genes were converted to human gene equivalents using ensemble numbers. The sequencing coverage and quality statistics for each sample are summarized in Supplementary Table S4.

### 2.7 Spatial transcriptome sequencing of the mouse model

The spatial transcriptome profile of mouse UTUC tissue was obtained using the NanoString GeoMx Digital Spatial Profiler (DSP) platform, with assays conducted at NanoString Technologies (Seattle, WA, USA). The DSP protocol, as previously described^19^ involved the following steps: an FFPE slide of mouse UTUC tissue was first deparaffinized, whereafter the mouse Whole Transcriptome Atlas probe reagent was applied. Subsequently, the slide was co‐incubated with fluorescently labeled antibodies to detect epithelial cells (pan‐cytokeratin), T cells (CD3), and macrophages (F4/80), together with DAPI to detect nuclei. Twenty‐one regions of interest (ROI), including two normal urothelium, two dysplasia, and 20 invasive UC sites, were selected based on histopathological morphology. Each ROI was illuminated with UV light, and the indexing oligonucleotides were collected in a 96‐well plate. Oligonucleotide probes were counted using a NanoString nCounter instrument to measure mRNA expression. The obtained mRNA expression data were normalized and analyzed using GeoMx DSP^19^ software and the iDEP platform.^18^ Gene ontology (GO) enrichment analysis was performed using the free‐access platform, Metascape^20^ An absolute FC cutoff value of 2.0 and *p*‐value <0.01 were used to identify the DEGs.

### 2.8 16S ribosomal RNA metagenome sequencing of the mouse model

Fecal samples were collected from dissected mice, immediately flash‐frozen, and stored at −80°C. DNA was extracted from fecal samples using the nucleic acid extraction system, PI‐1200 (Kurabo, Osaka, Japan). Each library was prepared for sequencing following the Illumina 16S Metagenomic Sequencing Library Preparation Guide, with primer set 27Fmod/338R targeting the V1–V2 region of the 16S rRNA genes. Amplicons were sequenced using 301‐bp paired‐end sequencing on a MiSeq system (Illumina). The paired‐end sequences obtained were then merged, filtered, and denoised using DADA2. Taxonomic assignment of the sequences was performed using the QIIME2 feature‐classifier plugin with the Greengenes 13_8 database. The QIIME2 pipeline (version 2020.2) was used as the bioinformatics environment to process all relevant raw sequencing data (available at https://qiime2.org).^21^ The sequencing coverage and quality statistics for each sample are summarized in Supplementary Tables S5 (each strain and sex), and S6 (dietary intervention).

### 2.9 Metabolome analysis of the mouse model

Fecal samples were collected from dissected mice, immediately flash‐frozen, and stored at −80°C. The fecal samples were combined with cold methanol, chloroform, Milli‐Q water, and zirconium beads, along with 10 μM 2‐morpholinoethanesulfonic acid (Tokyo Chemical Industry) and methionine sulfone (Tokyo Chemical Industry) for homogenization using an MS‐100 homogenizer (TOMY, Tokyo, Japan). After centrifugation at 15,000 × *g* for 10 min at 4°C, the supernatant was collected, evaporated, redissolved in ultrapure water, and stored at −80°C. Metabolite measurements were performed according to the Metabolites Method Package ver. 2, utilizing the semi‐targeted metabolomics approach developed by the Shimadzu Corporation (Kyoto, Japan). Samples were diluted five‐fold with Milli‐Q water for measurements, and metabolites were detected in the multiple reaction monitoring mode using an LCMS‐8060NX triple quadrupole mass spectrometer (Shimadzu) connected to a NexeraTMX3 system (Shimadzu). The results were normalized and analyzed using the free‐access platform, MetaboAnalyst,^22^ with peak areas adjusted for the weight of each stool sample. An absolute FC cutoff value of 1.5 and *p*‐value <0.05, as determined by Student's *t*‐test, were used to identify differentially expressed metabolites (DEMs). A *p*‐value <0.05 was considered statistically significant, as determined via the Wilcoxon test for comparisons between two groups. The result data for each sample are summarized in Supplementary Table S7.

### 2.10 Clinical samples

In the OU cohort, samples were collected from 74 patients who underwent radical total nephroureterectomy or needle biopsy at the Osaka University Hospital, as previously reported.^23^ Matched pairs of UTUC and AN samples were immediately frozen at −80°C and subsequently immersed in RNAlater for stable storage at −20°C. Genomic DNA and RNA were extracted using the DNA Mini (QIAGEN) and RNeasy mini kits (QIAGEN), respectively, following the manufacturer's protocol.

### 2.11 WES of human samples (OU cohort^23^)

WES of genomic DNA from 71 UTUC, along with matched germline samples (buffy coat DNA), was conducted using Agilent SureSelect XT Human All Exon V6 (Agilent Technologies) for target capture. Buffy coat DNA was isolated from blood lymphocytes using the QIAamp DNA Blood Mini Kit (QIAGEN), following the manufacturer's protocol. Raw sequence data were generated using the Illumina NovaSeq6000 platform (Illumina), employing a standard 150‐bp paired‐end read protocol at Macrogen Japan (Tokyo, Japan). FASTQ files were generated from the raw data using bcl2fastq2 conversion software. The Genomon pipeline (https://github.com/Genomon-Project/genomon-docs/tree/v2.0) was used to identify candidate somatic mutations by referencing the GRCh37/hg19 human genome. The candidate mutations in a tumor sample were identified based on the following criteria: (i) Fisher's exact test *p*‐value ≤0.1, (ii) presence of ≥5 variant reads in the tumor sample, (iii) variant allele frequency (VAF) in the tumor sample ≥0.08, and (iv) comparison of the VAF in the matched normal sample. The sequencing coverage and quality statistics for each sample are summarized in Supplementary Tables S8.

### 2.12 WTS of human samples (OU cohort^23^)

RNA integrity was verified using an Agilent 2100 Bioanalyzer with RNA Nano reagents (Agilent Technologies). RNA from 73 UTUC and 74 AN tissue samples was subjected to polyA+ selection and chemical fragmentation. The 100‐bp RNA fraction was utilized to construct cDNA libraries using the TruSeq Stranded mRNA Prep kit (Illumina), following the manufacturer's protocol. These paired‐end libraries were sequenced on an Illumina NovaSeq6000 platform using a standard 100‐bp paired‐end read protocol at Macrogen Japan.

Gene expression values for tumor samples were estimated from the RNA‐seq data using the Genomon pipeline (https://github.com/Genomon-Project/genomon-docs/tree/v2.0). Alignment was performed using the STAR aligner (v.2.5.2a) against the hg19 human genome. The BAM files, named Aligned.sortedByCoord.out.bam, which were generated via STAR, were utilized to quantify expression data using GenomonExpression. The sequencing coverage and quality statistics for each sample are summarized in Supplementary Table S9.

### 2.13 Statistical analysis

Statistical analyses were performed using R (version 4.2.2; https://www.r-project.org/), GraphPad Prism 9 (GraphPad Software, La Jolla, CA, USA), and JMP PRO16 software (SAS Institute, Cary, NC, USA). For data that passed the Shapiro–Wilk test for a normal distribution, statistical comparisons were made using an unpaired *t*‐test. For data that did not follow a normal distribution, comparisons were conducted using the Mann–Whitney U test. The Bonferroni correction was applied to adjust for multiple comparisons. Statistical significance was set at *p* <0.05. Graph bars represent the mean ± standard error.

## 3 RESULTS

### 3.1 UTUC developed only in female BALB/c mice

**FIGURE 1:**
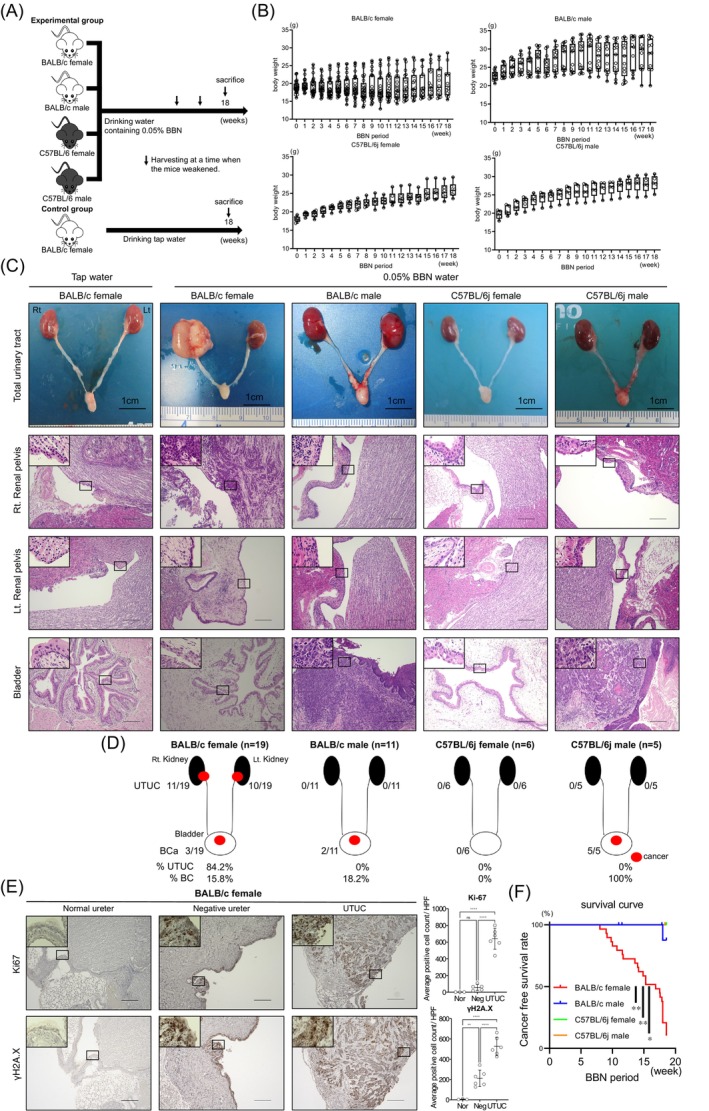
UTUC developed only in BALB/c female mice. (A) BALB/c and C57BL/6j male and female mice were administered 0.05% BBN and BALB/c female control mice were given tap water for 18 weeks. (BALB/c female: *n* = 29, BALB/c male: *n* = 13, C57BL/6j female: *n* = 6, C57BL/6j male: *n* = 5). (B) Changes in weight during the BBN administration period for each strain and sex (BALB/c female: *n* = 29, BALB/c male: *n* = 13, C57BL/6 female: *n* = 6, C57BL/6 female: *n* = 5). (C) Macroscopic and histopathological representations for each strain and sex. Scale bars for macroscopic images and histopathological images represent 1 cm and 200 μm, respectively. (D) Probability of UTUC or BC for each strain and sex. Ten BALB/c females and two BALB/c males were excluded due to unexpected death (BALB/c female: *n* = 19, BALB/c male: *n* = 11, C57BL/6 female: *n* = 6, C57BL/6 female: *n* = 5). (E) The left panel shows immunochemical staining for Ki‐67 and γH2A.X in a representative normal ureter from a BALB/c female mouse administered tap water, a macroscopically negative ureter, and UTUC from a BBN‐treated BALB/c female mouse. Scale bars represent 200 μm. The right graph shows the number of Ki‐67‐ and γH2A.X‐positive cells in the normal, negative ureter, and UTUC per high power fields (HPF) (normal ureter: *n* = 3, negative ureter: *n* = 5 or 6, UTUC: *n* = 6). Nor, normal ureter; Neg, negative ureter. Mann–Whitney U test was used. *p < 0.05. **p < 0.01. (F) Kaplan–Meier curves for each strain and sex. (BALB/c female: *n* = 29, BALB/c male: *n* = 13, C57BL/6 female: *n* = 6, C57BL/6 female: *n* = 5). The log‐rank test corrected with the Bonferroni method was performed. *p < 0.05. **p < 0.01. [Correction added on 10 January 2025, after first online publication: The Figure 1(A) has been replaced in this version.].

Experimental design is shown in Supplementary Figure S1. To identify the UTUC carcinogenic mouse strain and sex, BBN was administered to four different combinations of mouse strains and sexes (Figure 1A). Notably, only female BALB/c mice exhibited severe body weight loss (Figure 1B), although water consumption remained within the normal range (Supplementary Figure S2A).^24^ Histopathological examination revealed that 84.2% (16/19) of the female BALB/c mice developed invasive UTUC, whereas 15.8% had BC with concurrent UTUC (Figure 1C, D). Otherwise, C57BL/6j^11^, ^24^, ^25^, ^26^ or male BALB/c mice^24^ did not exhibit UTUC, and BC occurred in the males of some BALB/c^24^ and all C57BL/6j mice^11^ as previously reported (Figure 1C, D). The female C57BL/6j mice did not exhibit both BC and UTUC because the observation period was too short. In female BALB/c mice, macroscopically normal tissues of the renal pelvis treated with BBN showed increased DNA damage and cell proliferation compared to those in normal tissues, although to a lesser degree than in cancerous tissues (Ki‐67: normal vs. negative, *p* = 0.6652; normal vs. UTUC, *p* <0.0001; negative vs. UTUC, *p* <0.0001; γH2A.X: normal vs. negative, *p* = 0.0077; normal vs. UTUC, *p* <0.0001; negative vs. UTUC, *p* <0.0001) (Figure 1E). The female BALB/c mice bearing UTUC and treated with BBN experienced significantly earlier mortality than that in mice of other groups due to cancer progression and severe weight loss (BALB/c female vs. BALB/c male, *p* = 0.0002; BALB/c female vs. C57BL/6j female, *p* = 0.0005; BALB/c female vs. C57BL/6j male, *p* = 0.0062) (Figure 1F and Supplementary Figure S2B).

### 3.2 UTUC profile in female BALB/c mice

#### 3.2.1 Model profile in terms of somatic mutation

**FIGURE 2:**
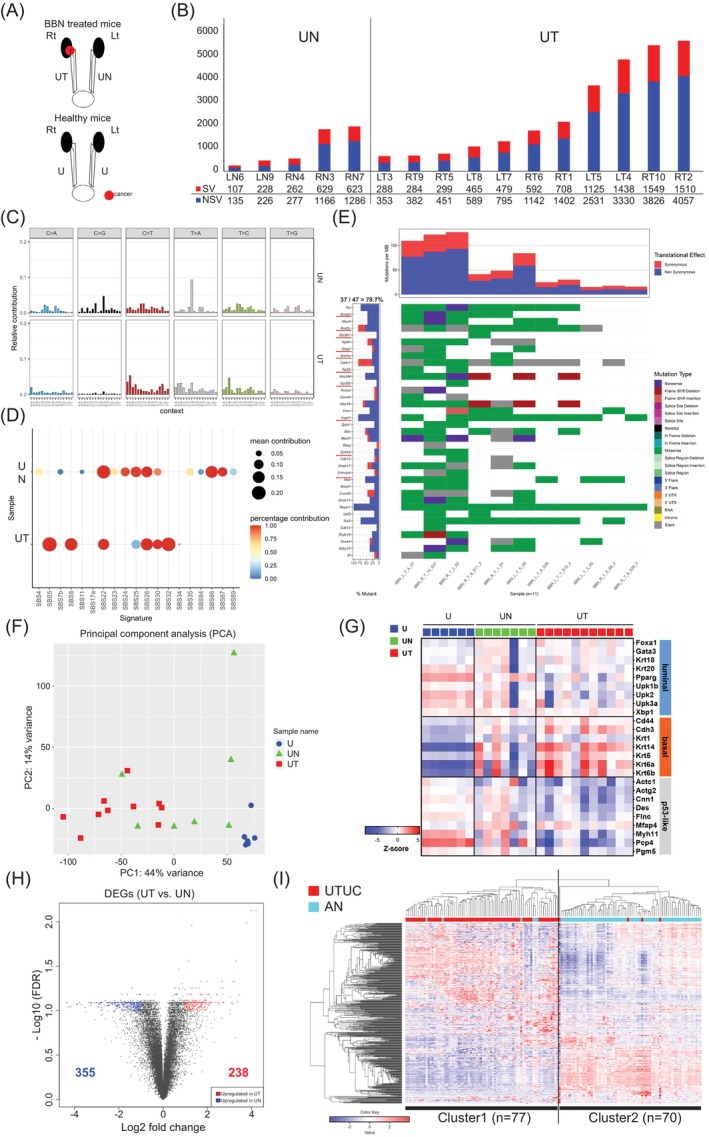
Profile of BALB/c female with UTUC using multi‐omics. (A) Sample preparation design from BALB/c female mice for whole exome sequencing (WES), whole transcriptome sequencing (WTS), and metabolome analysis. UT: ureter tumor indicates the cancerous side of the upper tract ureter of BBN‐treated BALB/c female mice. UN: ureter negative indicates the non‐cancerous side of the upper tract ureter of BBN‐treated BALB/c female mice. U: ureter indicates the normal ureter of healthy BALB/c female mice administered tap water. (B) The count of single nucleotide variants in the UN and UT samples, including synonymous variants (SV) and non‐synonymous variants (NSV) using WES data (UT: *n* = 11, UN: *n* = 5). Spleen samples for each individual were used for annotation. (C) Averaged single base substitution (SBS) signatures for UN (upper) and UT (lower) using WES data (UT: *n* = 11, UN: *n* = 5). (D) Absolute contribution of each SBS‐like pattern to UN (upper) and UT (lower) using WES data (UT: *n* = 11, UN: *n* = 5). (E) Mouse UT mutational plot using the gene list of the TCGA human UTUC cohort (UT: *n* = 11). The gene list contains over 10% frequent mutation genes in the TCGA human UTUC cohort. Red characters and red underlined genes are oncogenes. 37/47 genes were mutated in the model's UT sample. (F) Principal component analysis (PCA) of U, UN, and UT using WTS data (UT: *n* = 10, UN: *n* = 7, U: *n* = 6). (G) Molecular subtype by the Fantini‐Gene set using WTS data (UT: *n* = 10, UN: *n* = 7, U: *n* = 6). (H) The differentially expressed genes (DEGs) compared UT and UN using WTS data (UT: *n* = 10, UN: *n* = 7). Red dots indicate genes upregulated in UT (*n* = 238), and blue dots indicate genes upregulated in UN (*n* = 355). |FC| >2.0, FDR <0.1 were significant. FC, fold change. (I) Clustering analysis of human UTUC and adjacent normal upper urinary tract (AN) samples using 502 gene sets obtained from the model's DEGs analysis compared UT and UN. (UTUC: *n* = 74, AN: *n* = 73). Cluster 1 comprised 77 samples (71 UTUC samples and 6 AN samples) and cluster 2 comprised 70 samples (3 UTUC samples and 67 AN samples).

To confirm similarity to human UTUC genetically, we employed WES and WTS analyses. The analyzed samples are shown in Figure 2A. Upper urinary tract tumor samples from BBN‐treated mice (UT) exhibited nonsynonymous variants (NSVs), ranging from 382 to 4057 per sample, whereas the microscopically noncancerous side of the upper urinary tract of BBN‐treated mice (UN) presented with 135–1286 NSVs per sample (Figure 2B). Each chromosome was uniformly mutated in UT and UN (Supplementary Figure S3A).^15^ The SBS signatures in the UN samples were different from those in the previously reported BC model,^27^ particularly in the C > G and CTG > CAG mutations,^15^ whereas the SBS patterns of the UT samples were similar to those in the previously reported BC model(Figure 2C).^27^ When deconvoluting the mutational signatures of each organ, the UN signature was found to be similar to that of SBS25^15^, ^28^ and the UT signature mainly resembled the SBS5 typically associated with UC in humans (Supplementary Figure S3B)^28^ Further analysis of the contribution of SBS signatures in the UT and UN samples revealed that SBS22 was common to UT and UN, SBS5, SBS8, SBS30, and SBS32 were characteristic UT signatures, and SBS24, SBS25, SBS86, and SBS87 were distinctive to the UN signature (Figure 2D).^15^, ^28^

We evaluated the concordance between the gene mutations in our model and a list of genes with a high mutation frequency derived from two different cohorts: one from a public database^16^ and the other from the previously reported 74 patients with UTUC (OU cohort).^23^ We found that 78.7% (37/47) of the genes (Supplementary Table S10) aligned with those in the TCGA cohort (Figure 2E)^17^ and 77.8% (14/18; Supplementary Table S11) of the gene mutations in this model aligned with those in the OU cohort (Supplementary Figure S3C)^17^; *Fgfr3* or *Ras* mutations were absent, as in a previously reported BC model.^27^, ^29^

#### 3.2.2 Gene expression profile of the model

Using the 24,346 genes detected in our analysis, gene expression clustering and principal component analysis showed that UT and U belonged to distinct clusters, with UN clustered across UT and U (Figure 2F and Supplementary Figure S3D)^18^ When each sample was classified into basal, luminal, and p53‐like subtypes using the previously reported gene set^27^ UT exhibited a basal type similar to that of the previously reported BC model(Figure 2G).^27^ In addition to exhibiting a *Trp53* mutation and the absence of *Fgfr3* and *Ras* mutations (Figure 2E), the female BALB/c UTUC mouse model mimicked the C3 gene expression subtype of the TP53/MDM2 mutation or triple‐negative mutation subtypes of human UTUC.^8^

We also evaluated the gene expression similarities between the mouse model and humans. Compared to UN, we identified 238 upregulated and 355 downregulated genes in UT (FDR <0.1, FC ≥2.0; Figure 2H).^18^ We assessed the concordance in gene expression of the female BALB/c mice with UT and UN with those with UTUC and adjacent normal upper urinary tract (AN) of the OU cohort.^23^ In total, 488 genes (187 upregulated and 301 downregulated) among the 593 DEGs could be adjusted to human genes (Supplementary Table S12). We performed gene expression clustering analysis of the OU cohort^23^ using these 488 genes, dividing the 147 samples into two distinct clusters (Figure 2I).^18^ Cluster 1 comprised 92% of UTUC samples (71/77), whereas Cluster 2 comprised 96% of AN samples (67/70).

### 3.3 Female BALB/c UTUC mice have genetic spatial heterogeneity mimicking human prognosis

#### 3.3.1 Genetic spatial heterogeneity of the female BALB/c UTUC mouse model

**FIGURE 3:**
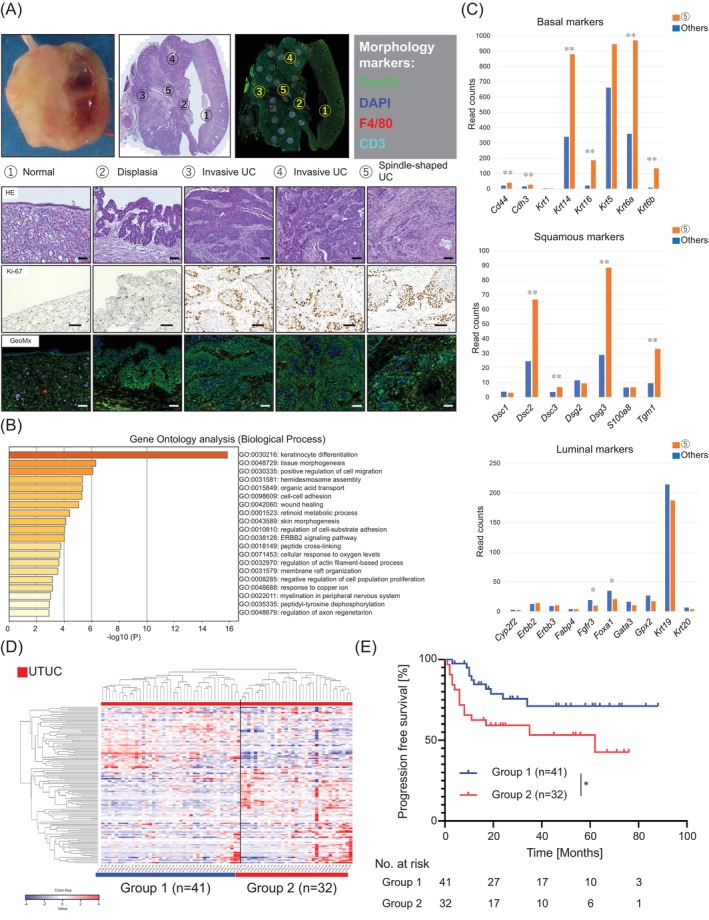
BALB/c female UTUC mimics human UTUC in multiple aspects. (A) A representative sample was analyzed by spatial transcriptome sequencing. (Upper) Overall appearance of macroscopic, histopathological, and fluorescence immunostaining. (Lower) Representative microscopic appearance of histopathological, Ki‐67 staining, and fluorescence immunostaining from each region of interest (ROI). ROI ①: Normal urothelium (*n* = 2), ROI ②: Dysplasia (*n* = 2), ROI ③: Invasive UC (*n* = 12), ROI ④: Invasive UC (*n* = 3), ROI ⑤: Invasive UC with spindle‐shaped cell (*n* = 5). In the fluorescence immunostaining, Green: PanCK, Blue: DAPI, Red: F4/80, Light blue: CD3. Scale bars represent 50 μm. (B) GO enrichment analysis in ⑤ versus ②–④ showed that ⑤ was enriched of GOs involved in cancer malignancy, such as keratinocyte differentiation, tissue morphogenesis, and cell migration and adhesion. ROI ①: Normal urothelium (*n* = 2), ROI ②: Dysplasia (*n* = 2), ROI ③: Invasive UC (*n* = 12), ROI ④: Invasive UC (*n* = 3), ROI ⑤: Invasive UC with spindle‐shaped cell (*n* = 5). (C) Evaluation of gene expression characteristic of human UTUC molecular subtypes in comparison ⑤ versus ②–④ revealed that basal markers (upper) and squamous markers (middle) were upregulated in ⑤, while luminal markers (lower) were downregulated in ⑤. Mann–Whitney U test was used. *p <0.05. **p <0.01. (D) Clustering analysis of patients with UTUC (*n* = 73) using 123 gene sets obtained from the model's DEGs analysis compared ROI ⑤ and ROI ①, ② and ROI ③, ④. Group 2 shows significantly upregulated genes from ROI ⑤. (E) Progression‐free survival (PFS) of Groups 1 and 2 analyzed by the Kaplan–Meier method. The log‐rank test was used. *p <0.05.

The advantage of this model lies in its use of non‐genetically modified mice. Then, we verified whether this model mimics human UTUC with strong heterogeneity using spatial transcriptome sequencing. The analysis was performed using a representative sample to verify heterogeneity within the mouse model (Figure 3A).^19^ Within a single tumor, there were three microscopically different sections with high cell proliferation (①: normal urothelial site, ②: urothelial dysplasia site, ③, ④: non‐spindle‐shaped invasive UC site, and ⑤: spindle‐shaped invasive UC site). Gene Ontology biological process (GOBP) enrichment analysis in ⑤ versus ③, ④ indicated that ⑤ was enriched with GOBPs involved in cancer malignancy, such as epithelial cell differentiation, tissue mutation, and cell metastasis and adhesion (Figure 3B)^20^ Evaluation of the gene expression characteristics of ⑤ using UTUC molecular subtypes^8^ revealed that basal and squamous markers were upregulated, whereas luminal and FGFR3 markers were downregulated in ⑤ (upregulated in ⑤: *Cd44*, *p* < 0.01; *Cdh3*, *p* < 0.01; *Krt14*, *p* < 0.01; *Krt16*, *p* < 0.01; *Krt6a*; *p* < 0.01; *Krt6b*, *p* < 0.01; *Dsc2, p <* 0.01; *Dsc3, p* < 0.01; *Dsg3*, *p* < 0.01; *Tgm1*, *p* < 0.01; *Ptpn13*, *p* < 0.05; downregulated in ⑤: *Fgfr3*, *p* < 0.05, *Foxa1*, *p* < 0.05; Figure 3C and Supplementary Figure S4A).

#### 3.3.2 Genetic spatial heterogeneity reflects the prognosis of patients with UTUC

We examined whether the genetic spatial heterogeneity of the mouse model reflected the prognosis of patients with UTUC. Gene expression of PanCK‐positive cells was compared between the ⑤ and ①, ② sites and between the ⑤ and ③, ④ sites (*p* < 0.05, FC ≥2.0; Supplementary Figure S4B).^18^ We identified 144 DEGs common in ⑤ versus ①, ② and ⑤ versus ③, ④, with 123 genes that could be translatable to human genes (Supplementary Figure S4C). Seventy‐three patients with UTUC from the OU cohort^23^ were classified into two groups using these 123 gene sets (Figure 3D),^18^ with patients classified into Group 2 (*n* = 32) exhibiting a significantly poorer prognosis than those in Group 1 (*n* = 41) (*p* < 0.0326; Figure 3E). This heterogeneity suggests that the mouse model accurately mirrors the real‐world scenario of human UTUC, which is characterized by heterogeneous histology and molecular biology.^30^

### 3.4 The mouse model exhibits increased inflammatory gene expression in the upper urinary tract

#### 3.4.1 Inflammatory gene expression is upregulated in UT

**FIGURE 4:**
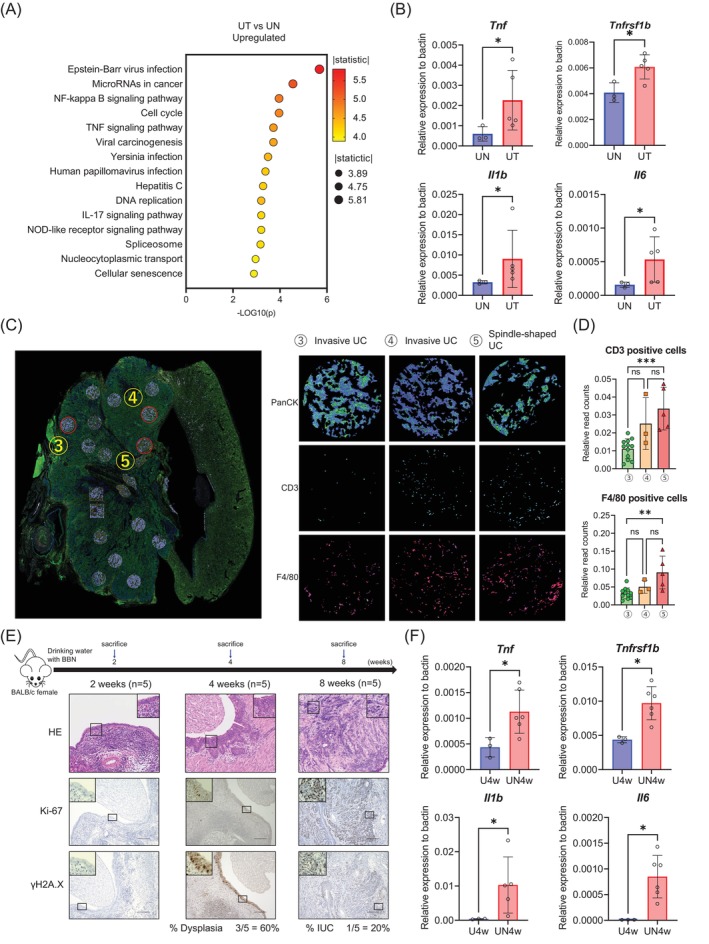
Elevated inflammatory gene expression in the upper urinary tract is a feature of this model. (A) Pathway enrichment analysis of UT versus UN based on the KEGG database using WTS data (UT: *n* = 10, UN: *n* = 7). (B) Expression of four *Tnf*‐related inflammatory genes (*Tnf*, *Tnfrsf1b*, *Il1b*, and *Il6*) in UN and UT samples determined by qPCR (UN: *n* = 3, UT: *n* = 5). The Mann–Whitney U test was used. *p < 0.05. (C) Representative region of each ROI ③, ④, and ⑤; the same image of Figure 3A. ROI ① and ② were removed from assessment because CD3‐ or F4/80‐positive cells were not detected. Blue: PanCK. Green: CD3. Red: F4/80. (D) Comparative analysis of relative read counts of CD3 (upper) or F4/80 (lower) positive cells across ROI ③, ④, and ⑤ (ROI ③: *n* = 12, ROI ④: *n* = 3, and ROI ⑤: *n* = 5). The Kruskal–Wallis test was used. n.s. means not significant. **p <0.01. ***p <0.005. (E) Experimental schema for varying BBN exposure durations (2‐, 4‐, and 8‐weeks) in BALB/c females, and microscopic and immunochemical staining of representative samples. (2 weeks: *n* = 5, 4 weeks: *n* = 5, 8 weeks: *n* = 5.) Scale bars represent 200 μm. (F) Four *Tnf*‐related inflammatory genes (*Tnf*, *Tnfrsf1b*, *Il1b*, and *Il6*) expression by qPCR using visibly normal ureter of BALB/c female treated 4 weeks BBN (UN4: *n* = 5–6) and healthy ureter of BALB/c female given 4 weeks tap water (U4: *n* = 3). Mann–Whitney U test was used. *p <0.05.

Subsequently, we conducted pathway enrichment analysis to understand the cancer characteristics of this model. GO enrichment analysis highlighted the enrichment of processes related to immunity and defense in UT versus UN (Supplementary Figure S5A).^18^ Kyoto Encyclopedia of Genes and Genomes pathway enrichment analyses revealed that the TNF signaling pathway was more enriched in UT than in UN (Figure 4A).^18^, ^31^, ^32^ We validate this by assessing the mRNA expression levels of inflammatory genes *Tnf*, *Tnfrsf1b*, *Il1b*, and *Il6*. All genes were upregulated in UT than in UN (*p* = 0.0369; Figure 4B).

#### 3.4.2 T cells and macrophages increasingly infiltrate the basal site

In response to these findings, we focused on CD3‐ or F4/80‐positive cells infiltrating each cancer site using spatial transcriptome sequencing (Figure 4C). The read counts of both CD3‐positive T cells and F4/80‐positive macrophages within ⑤ were significantly higher than those in ③ (CD3, *p* < 0.0026; F4/80, *p* < 0.0441; Figure 4D).^19^ Furthermore, four genes expressed in CD3‐positive T cells, including *Mgp*, which is linked to T cell exhaustion through the NF‐kB pathway^33^ were upregulated in ⑤ (*p* < 0.01, FC ≥2.0; Supplementary Figure S5B), and 242 genes of F4/80‐positive macrophages were differentially expressed between ③ and ④, ⑤ (*p* < 0.01, FC ≥2.0; Supplementary Figure S5C).^18^ GOBP enrichment analysis in ③ to ④, ⑤ of F4/80‐positive macrophages showed that ④, ⑤ were enriched with GOBPs involved in the immune system (Supplementary Figure S5D).^20^

#### 3.4.3 Inflammatory gene expression is upregulated in the upper urinary tract before carcinogenesis

We conducted a short‐term BBN administration experiment to evaluate whether the increase in inflammatory gene expression, characteristic of this model, occurs prior to carcinogenesis. Further evaluation of the temporal changes in the ureteral tissues treated with BBN showed that 2 weeks of treatment led to immune cell infiltration into the stroma; 4 weeks of treatment led to dysplasia in 60% of samples (3/5); and 8 weeks of treatment led to invasive UTUC in 20% of samples (1/5; Figure 4E). Immunostaining assessment of DNA damage and cell proliferation revealed no changes after 2 weeks of BBN treatment. However, DNA damage and cell proliferation increased after 4 weeks of treatment with BBN, indicating that carcinogenesis might begin invisibly at approximately 4 weeks (Figure 4E). We assessed the same four genes in BBN‐treated female BALB/c mice at 4 weeks and found that BBN treatment increased the expression of inflammatory genes in the upper urinary tract (*Tnf*, *p* = 0.0476; *Tnfrsf1b*, *p* = 0.0238; *Il1b*, *p* = 0.0357; *Il6*, *p* = 0.0238; Figure 4F).

### 3.5 Dietary intervention alters the gut microbiota of female BALB/c mice

**FIGURE 5:**
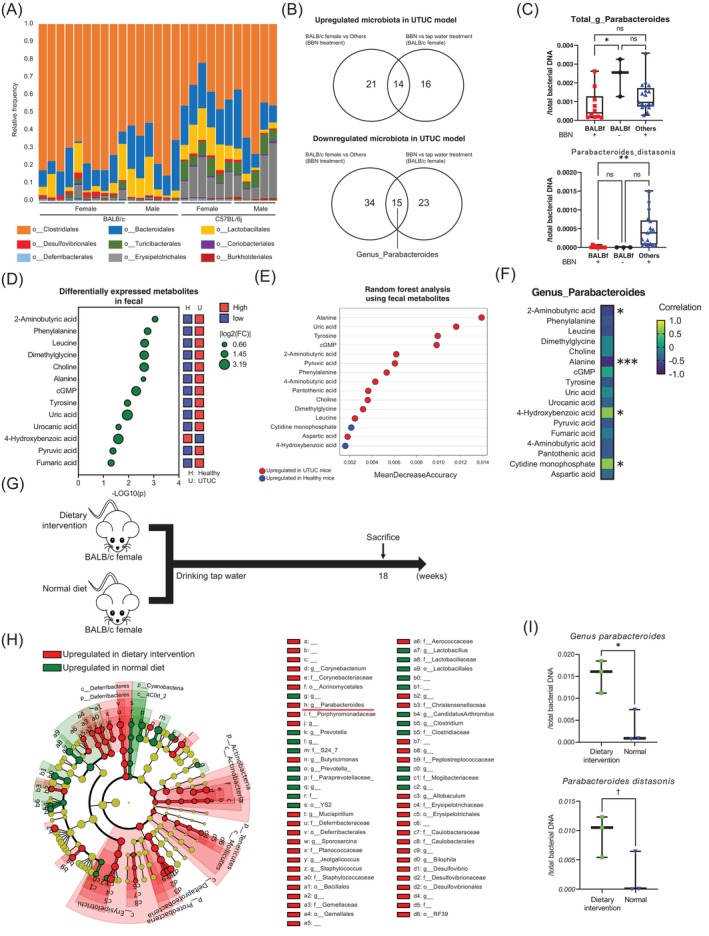
Dietary intervention increases the abundance of *Parabacteroides distasonis* in BALB/c female mice feces. (A) Taxa‐bar plot at the order level (o) from 16S rRNA‐seq data. BALB/c female treated with BBN for 18 weeks: *n* = 10. BALB/c male treated with BBN for 18 weeks: *n* = 6. C57BL/6j female treated with BBN for 18 weeks: *n* = 6. C57BL/6j male treated with BBN for 18 weeks: *n* = 5). (B) Identification of gut microbiota more and less abundant in BALB/c females than in the others (BALB/c male, C57BL/6j female and male) treated with BBN for 18 weeks, as well as, in BALB/c females treated with BBN and those administered with tap water (BALB/c female treated with BBN: *n* = 10, Others treated with BBN: *n* = 17, BALB/c female treated without BBN: *n* = 3). 15 microbes, including *the genus Parabacteroides*, showed reduced abundances in BALB/c females treated with BBN for 18 weeks. (C) The relative abundance of *genus Parabacteroides* was significantly reduced in BALB/c females treated with BBN for 18 weeks compared to BALB/c females without BBN treatment (upper). The relative abundance of *Parabacteroides distasonis* was significantly reduced in BALB/c female treated with BBN for 18 weeks compared to others (BALB/c female treated with BBN: *n* = 10, Others treated with BBN: *n* = 17, BALB/c female treated without BBN: *n* = 3). Kruskal–Wallis test was used. n.s. means not significant. *p <0.05. **p <0.01. (D) Differentially expressed metabolites between BALB/c female treated with tap water (H: healthy) and with BBN water (U: UTUC) in fecal (UTUC: *n* = 10, Healthy: *n* = 3). Student's *t*‐test was used. |FC| >1.5, *p* <0.05 were significant. (E) Random forest analysis of fecal samples from UTUC and healthy BALB/c female mice (UTUC: *n* = 10, Healthy: *n* = 3). Red bubble: Upregulated in UTUC mice. Blue bubble: upregulated in Healthy mice. (F) Correlation analysis between differentially expressed fecal metabolites and *genus Parabacteroides* in BALB/c female treated with or without BBN (*n* = 13). An analysis was performed using the Pearson product–moment correlation coefficient. *p <0.05. **p <0.01. ***p <0.005. (G) Experimental design for BALB/c female with dietary intervention (*n* = 3) or normal diet (*n* = 3). (H) Linear discriminant analysis effect size (LEfSe). Comparison of the abundance of gut microbiota between BALB/c females with dietary intervention (*n* = 3) versus normal diet (*n* = 3) in circle plot (left) and bar chart (right). Red underlined microbiota indicates the *genus Parabacteroides*. Green bar: upregulated in the normal diet group. Red bar: upregulated in the dietary intervention group. k, kingdom; p, phylum; c, class; o, order; f, family; g, genus; s, species. (I) The relative abundance of *genus Parabacteroides* was significantly increased in BALB/c females with dietary intervention (*n* = 3) than in the normal diet group (*n* = 3, upper). The relative abundance of *Parabacteroides distasonis* was significantly increased in BALB/c females in the dietary intervention (*n* = 3) than in the normal diet group (*n* = 3, lower). Mann–Whitney U test was used. †*p* <0.1. *p <0.05.

Subsequently, we assessed the gut microbiota and metabolites, which have been highlighted for their associations with inflammation and cancer recently.^34^, ^35^, ^36^, ^37^ The *α*‐diversity of gut microbiota showed no difference between female BALB/c mice and those in other groups treated with BBN (Supplementary Figure S6A) and tended to be higher in female BALB/c mice treated with BBN compared to those treated with tap water (Supplementary Figure S6B).^21^ Taxa bar plots of the gut microbiota varied between the strains and sexes treated with BBN (Figure 5A)^21^ Linear discriminant analysis effect size (LEfSe) analysis identified 14 more abundant and 15 less abundant microbes in female BALB/c mice than in other groups treated with BBN, as well as, female BALB/c mice treated with or without BBN (Figure 5B and Supplementary Figure S6C, D).^21^ Among the microbes with reduced abundances in the UTUC model, we focused on the genus *Parabacteroides*, which is the upper genus of *Parabacteroides distasonis* and is known to reduce inflammatory cytokine expression.^34^, ^35^, ^36^, ^37^ We found that *P. distasonis* was virtually absent in the feces of female BALB/c mice, both in those with and without BBN administration (Figure 5C). Next, we evaluated 72 metabolites in feces using metabolome analysis. Partial least squares‐discriminant analysis of stool metabolites showed a distinct separation between UTUC and healthy BALB/c mice (Supplementary Figure S6E)^22^ The levels of 12 metabolites, including alanine, were increased and one decreased in the fecal samples of female BALB/c mice treated with BBN (*p* < 0.05, FC ≥1.5; Figure 5D).^22^ Random forest analysis revealed that alanine was the most distinguishable factor in the stool samples of female BALB/c mice with UTUC compared with that of healthy female BALB/c mice (Figure 5E).^22^ Moreover, correlation analysis between the gut microbiota and fecal metabolites revealed that alanine had the strongest negative correlation with the genus *Parabacteroides* (Figure 5F and Supplementary Figure S6F). We then assessed whether an alanine‐free dietary intervention (Supplementary Table S1) altered the gut microbiota (Figure 5G). The alanine‐free dietary intervention increased the abundance of both genus *Parabacteroides* and *P. distasonis* (Figure 5H, I).

### 3.6 Dietary intervention and subsequent gut microbiota alteration can prevent UTUC carcinogenesis in female BALB/c mice

**FIGURE 6:**
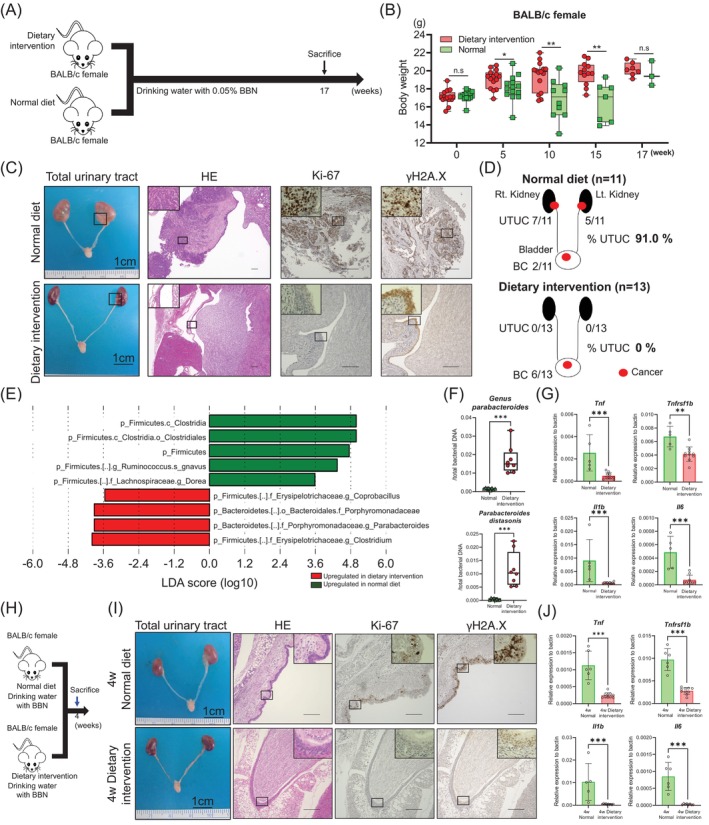
Dietary intervention can prevent UTUC carcinogenesis in BALB/c female mice. (A) Experimental design for BBN administration for BALB/c female with dietary intervention or normal diet for 17 weeks (dietary intervention group: *n* = 15, normal diet group: *n* = 14). (B) Weight changes during BBN administration in BALB/c females with dietary intervention or normal diet (dietary intervention group: *n* = 15, normal diet group: *n* = 14). Mann–Whitney U test was used. n.s. means not significant. *p < 0.05. **p < 0.01. (C) Macroscopic and histopathological appearance of BALB/c female treated with BBN on dietary intervention or normal diet for 17 weeks. Scale bars of macroscopic images and histopathological images represent 1 cm and 200 μm, respectively. (D) The frequency of UTUC in each group. In BALB/c females with normal diet (upper), 63.6% (7/11) had UTUC in the right upper urinary tract, 45.5% (5/11) had UTUC in the left upper urinary tract, and 18.2% (2/11) had UTUC bilaterally. Thus, the carcinogenic rate of UTUC is 91.0% (7 + 5 − 2/11). 18.2% (2/11) had BC. In BALB/c females with dietary intervention (lower), 0.0% (0/13) had UTUC in the right upper urinary tract and 0.0% (0/13) had UTUC in the left upper urinary tract. Thus, the carcinogenic rate of UTUC is 0.0% (0/13). 46.2% (6/13) had BC. (E) Linear discriminant analysis effect size (LEfSe) identified bacteria with common changes in the comparison with UTUC versus others treated with BBN from Figure 5B and BALB/c female with dietary intervention versus normal diet treated with BBN (dietary intervention group: *n* = 8, normal diet group: *n* = 8). Green bar: upregulated in BALB/c females with a normal diet. Red bar: upregulated in BALB/c female with an alanine‐free diet. p, phylum; c, class; o, order; f, family; g, genus; s, species. (F) The relative abundance of both *genus Parabacteroides* (upper) and *Parabacteroides distasonis* (lower) were significantly increased in BALB/c females treated with BBN on dietary intervention (*n* = 8) compared to normal diet (*n* = 8, upper). Mann–Whitney U test was used. ***p < 0.005. (G) Expression of four *Tnf*‐related inflammatory genes (*Tnf*, *Tnfrsf1b*, *Il1b*, and *Il6*) were assessed by qPCR in UT of BALB/c female treated with BBN on normal diet, and ureter of BALB/c female treated with BBN on dietary intervention (normal diet group: *n* = 5, dietary intervention group: *n* = 10). The Mann–Whitney U test was used. **p < 0.01. ***p < 0.005. (H) Experimental design for BBN administration for BALB/c female with dietary intervention or normal diet for 4 weeks (dietary intervention group 4: *n* = 6, normal diet group 4: *n* = 5). (I) Macroscopic and histopathological appearance of BALB/c female treated with BBN on dietary intervention or normal diet for 4 weeks. Scale bars of macroscopic images and histopathological images represent 1 cm and 200 μm, respectively. (J) Expression of four *Tnf*‐related inflammatory genes (*Tnf*, *Tnfrsf1b*, *Il1b*, and *Il6*) were assessed by qPCR in UN of BALB/c female treated with BBN on a normal diet, and ureter of BALB/c female treated with BBN on dietary intervention (normal diet group 4: *n* = 5–6, dietary intervention group 4: *n* = 10). The Mann–Whitney U test was used. ***p < 0.005.

Finally, we evaluated the impact of the gut microbiota remodeled by this dietary intervention on the BBN‐induced UTUC mouse model (Figure 6A). Dietary intervention protected female BALB/c mice from severe weight loss, which was observed in the normal diet group (*p* < 0.05–<0.001; Figure 6B). UTUC carcinogenesis was prevented in 91.0% (10/11) to 0% (0/13) of female BALB/c mice through dietary intervention (*p* < 0.0001; Figure 6C, D). Immunostaining results showed that cell proliferation was decreased in response to the dietary intervention, although DNA damage occurred regardless of the diet used (Figure 6C). We then compared the gut microbiota of the dietary intervention and normal diet groups. The dietary intervention group showed a significant decrease in *α*‐diversity (Chao1, *p* = 0.0002; Shannon diversity index, *p* = 0.0104; Simpson's diversity index, *p* = 0.0499; OTUs, *p* = 0.0002; Supplementary Figure S7A). The *β*‐diversity and taxa bar plot showed substantial changes following dietary intervention (Supplementary Figure S7B, C).^21^ LEfSe analysis revealed that of the 29 microbes specific to the UTUC mouse model (Figure 5B), the abundances of five were increased and four, including the genus *Parabacteroides*, were decreased and were significantly associated with UTUC carcinogenesis (Figure 6E and Supplementary Figure S7D).^21^ In the BBN treatment condition, this dietary intervention significantly and consistently increased the abundance of both the genus *Parabacteroides* and *P. distasonis* (Figure 6F). The expression of *Tnf*‐associated inflammation‐related genes was significantly downregulated in the upper urinary tract of the dietary intervention group (*Tnf*, *p* = 0.0007; *Tnfrsf1b*, *p* = 0.0047; *Il1b*, *p* = 0.0007; *Il6*, *p* = 0.0010; Figure 6G). To investigate the downregulation of these inflammatory genes before carcinogenesis, we assessed their expression after 4 weeks of BBN treatment (Figure 6H). Female BALB/c mice with or without dietary intervention showed no UTUC at 4 weeks after BBN treatment (Figure 6I). The dietary intervention group exhibited reduced BBN treatment‐induced DNA damage and cell proliferation (Figure 6I). The expression of *Tnf*‐associated inflammation‐related genes was significantly and consistently downregulated in the upper urinary tract of the dietary intervention group before carcinogenesis (*Tnf*, *p* = 0.0002; *Tnfrsf1b*, *p* = 0.0002; *Il1b*, *p* = 0.0007; *Il6*, *p* = 0.0007; Figure 6J).

## 4 DISCUSSION

To the best of our knowledge, this is the first study to successfully induce UTUC over BC using BBN in an animal model. Previous reports concluded that female BALB/c mice were unsuitable for the BBN‐induced UC carcinogenic model because of their early mortality^38^; however, our study revealed that the cause of death in female BALB/c mice was attributed to UTUC carcinogenesis. In humans, the prognosis of UC is poorer in females than in males, the reasons for which have not yet been identified.^39^, ^40^, ^41^ Therefore, this model may be useful for gaining new insights into the mechanisms by which sex differences affect the prognosis of UC in clinical practice.

This mouse model genetically mirrors human UTUC. In particular, the molecular subtypes from the WTS indicated highly malignant basal‐type gene expression in our model, suggesting that it mimics UTUC with a poor prognosis. This non‐engineered mouse model possesses histological, immunological, and molecular heterogeneity within a single tumor tissue, with gene expression reflecting the prognosis of human UTUC and T‐cell exhaustion. Therefore, we believe that this model is highly suitable for drug‐testing experiments involving immune checkpoint inhibitors.

In the microbiome analysis, we identified four microbes, including the genus *Parabacteroides*, to be associated with UTUC carcinogenesis. Furthermore, *P. distasonis* was less abundant in the female BALB/c mouse gut microbiome. Recent studies have highlighted that *P. distasonis* might suppress multiple human benign diseases and colorectal cancer by reducing inflammatory cytokine expression.^34^, ^35^, ^36^, ^37^ In our study, dietary intervention, mainly without alanine, increased the relative abundance of *P. distasonis* in the feces of female BALB/c mice and reduced inflammatory cytokine expression in the upper urinary tract before carcinogenesis. While we could not demonstrate a direct link between dietary intervention, *P. distasonis*, and the prevention of UTUC carcinogenesis, our findings suggest an essential role for changes in the intestinal environment in response to dietary alterations in the suppression of UTUC carcinogenesis. Future studies should elucidate the mechanisms underlying the alteration of the intestinal environment in response to the suppression of UTUC carcinogenesis, including through dietary alterations, as demonstrated in our study. Additionally, it is important to investigate why this phenomenon is absent in BC through detailed analyses of specific immunological reactions and the microbiome. Nevertheless, our results suggest that the regulation of inflammatory cytokines through the gut microbiota plays an important role in UTUC carcinogenesis.

In conclusion, we established a novel BBN‐induced mouse model of UTUC carcinogenesis that reflects several aspects of human UTUC. Therefore, this model may contribute to future studies on UTUC from a biological perspective.

## AUTHOR CONTRIBUTIONS

**Akinaru Yamamoto:** Conceptualization; methodology; data curation; investigation; validation; formal analysis; resources; project administration; visualization; funding acquisition; writing – original draft; writing – review and editing; software. **Atsunari Kawashima:** Conceptualization; writing – review and editing; writing – original draft; funding acquisition; project administration; resources; data curation; visualization; methodology; validation; formal analysis; investigation; software. **Toshihiro Uemura:** Writing – review and editing; resources. **Kosuke Nakano:** Resources; writing – review and editing; methodology; data curation; validation. **Makoto Matsushita:** Methodology; resources; writing – review and editing. **Yu Ishizuya:** Conceptualization; methodology; visualization; writing – review and editing; resources; data curation; validation. **Kentaro Jingushi:** Writing – review and editing; formal analysis; validation. **Hiroaki Hase:** Methodology; visualization; writing – review and editing; data curation; formal analysis; validation; funding acquisition. **Kotoe Katayama:** Methodology; validation; writing – review and editing. **Rui Yamaguchi:** Methodology; validation; writing – review and editing. **Nesrine Sassi:** Writing – review and editing. **Yuichi Motoyama:** Validation; writing – review and editing; formal analysis; methodology. **Satoshi Nojima:** Validation; formal analysis; writing – review and editing. **Masashi Mita:** Writing – review and editing. **Tomonori Kimura:** Writing – review and editing. **Daisuke Motooka:** Methodology; validation; formal analysis; data curation; writing – review and editing. **Yuki Horibe:** Resources; writing – review and editing. **Yohei Okuda:** Resources; writing – review and editing. **Toshiki Oka:** Resources; writing – review and editing. **Gaku Yamamichi:** Resources; writing – review and editing. **Eisuke Tomiyama:** Resources; writing – review and editing. **Yoko Koh:** Resources; writing – review and editing. **Yoshiyuki Yamamoto:** Resources; writing – review and editing. **Taigo Kato:** Resources; writing – review and editing. **Koji Hatano:** Resources; writing – review and editing. **Motohide Uemura:** Resources; writing – review and editing. **Seiya Imoto:** Supervision; writing – review and editing. **Hisashi Wada:** Supervision; writing – review and editing. **Eiichi Morii:** Supervision; writing – review and editing. **Kazutake Tsujikawa:** Supervision; writing – review and editing. **Norio Nonomura:** Supervision; writing – review and editing.

## FUNDING INFORMATION

The authors of the study were supported by the following grants: Japan Society for the Promotion of Science (C) (19K09709) (AK). Japan Society for the Promotion of Science Grant‐in‐Aid for Research Activity Start‐up (21K20968) (AY). Japan Society for the Promotion of Science (C) (22K09523) (AY). Japan Society for the Promotion of Science (B) (22H03213) (AK). AMED under Grant Number (JP22ym0126809i0002) (AK). Japan Society for the Promotion of Science Grant‐in‐Aid for Challenging Research (Pioneering) (22K18398) (AK). Research Support Project for Life Science and Drug Discovery (Basis for Supporting Innovative Drug Discovery and Life Science Research [BINDS]) from AMED under Grant Number (JP23ama121054) (HH).

## CONFLICT OF INTEREST STATEMENT

The authors declare that they have no competing interests.

## ETHICS STATEMENT

This study adhered to all the applicable ethical regulations and was approved by the Ethics Committee of Osaka University (#13397‐22). Written informed consent was obtained from all participants. All animal procedures were approved by the Osaka University Animal Research Committee (approval numbers: Douyaku R04‐11, Douyaku R04‐12, and Douyaku R05‐23) and performed in accordance with the relevant regulatory standards.

## Supporting information


**Table S3:** The sequencing coverage and quality statistics of mouse whole exome sequencing for each samples.

**Table S4:** The sequencing coverage and quality statistics of mouse whole transcriptome sequencing for each sample.

**Table S5:** The sequencing coverage and quality statistics of mouse 16S rRNA sequensing for each sample and sex.

**Table S6:** The sequencing coverage and quality statistics of mouse 16S rRNA sequensing for each sample with dietary intervention.

**Table S7:** The result data of mouse fecal metabolome analysis for each sample.

**Table S8:** The sequencing coverage and quality statistics of human whole exome sequencing for each samples.

**Table S9:**The sequencing coverage and quality statistics of human whole transcriptome sequencing for each sample.

**Table S10:** Mutational gene list of human whole exome sequencing in TCGA cohort.

**Table S11:** Mutational gene list of human whole exome sequencing in OU cohort.

**Table S12:**Differentially expressed gene list between mouse UT and UN.

**Data S1.** Supporting Information.

## Data Availability

The raw WES and RNA‐seq data from patient samples and data generated in this study are available in SRA under accession number PRJNA1151674 (WES) and GEO under accession number GSE275638 (RNA‐seq). The raw RNA‐seq, WES and 16S rRNA‐seq data from the mouse model and data generated in this study are available in GEO under accession number GSE274057 (RNA‐seq) and SRA under accession number PRJNA1146925 (WES), PRJNA1145594(16S rRNA‐seq) and PRJNA1145661(16S rRNA‐seq). The semi‐targeted metabolomics data are listed in Supplementary Table S7. All data pertinent to this study are included in the main text or the supplementary materials. The R code to reproduce the results and figures is publicly available at Bioconductor Mutationalpatterns (https://bioconductor.org/packages/release/bioc/html/MutationalPatterns.html), and GenVisR (https://www.bioconductor.org/packages/release/bioc/html/GenVisR.html). Any additional data supporting the findings are available from the corresponding author upon reasonable request.
