# Supplementary material for: A novel mouse model of upper tract urothelial carcinoma highlights the impact of dietary intervention on gut microbiota and carcinogenesis prevention despite carcinogen exposure
Source: Int J Cancer. 2024 Dec 18;156(7):1439–56. doi: 10.1002/ijc.35295 (PMC11789449; doi:10.1002/ijc.35295)
Supplement: Supplementary file 11 — Data S1. Supporting Information. [file IJC-156-1439-s010.pdf]

Supplementary Material

A novel mouse model of upper tract urothelial carcinoma highlights the impact of dietary intervention on gut microbiota and carcinogenesis prevention despite carcinogen exposure

Akinaru Yamamoto, Atsunari Kawashima, Toshihiro Uemura, Kosuke Nakano, Makoto Matsushita, Yu Ishizuya, Kentaro Jingushi, Hiroaki Hase, Kotoe Katayama, Rui Yamaguchi, Nesrine Sassi, Yuichi Motoyama, Satoshi Nojima, Masashi Mita, Tomonori Kimura, Daisuke Motooka, Yuki Horibe, Yohei Okuda, Toshiki Oka, Gaku Yamamichi, Eisuke Tomiyama, Yoko Koh, Yoshiyuki Yamamoto, Taigo Kato, Koji Hatano, Motohide Uemura, Seiya Imoto, Hisashi Wada, Eiichi Morii, Kazutake Tsujikawa, and Norio Nonomura

Table of Contents:

Supplementary Tables S1 to S12,

Supplementary Tables S3 to S12 are available in separate files

Supplementary Figures S1 to S7

Supplementary Table. S1. Components of normal and alanine free diet.

Nutrition components	unit	MF	AIN-93G modified, no alanine added
Moisture	g	8.1	9.0
Crude protein	g	23.2	17.8
Crude fat	g	4.9	7.1
Crude ash	g	5.9	2.7
Crude fiber	g	3.3	4.9
NFE	g	54.7	58.5
Total calories	kcal	355.7	369.2
Ca	g	1.04	0.49
P	g	0.81	0.16
Mg	g	0.24	0.05
Na	g	0.21	0.10
K	g	0.99	0.35
Isoleucine	g	0.92	0.921
Leucine	g	1.77	1.595
Lysine hydrochloride	g	1.27	1.705
Methionine	g	0.43	0.510
Cystine	g	0.36	0.382
Phenylalanine	g	1.06	0.871
Tyrosine	g	0.74	0.953
Threonine	g	0.89	0.707
Tryptophan	g	0.3	0.214
Valine	g	1.11	1.151
Histidine	g	0.62	0.510
Arginine	g	1.47	0.625
Alanine	g	1.19	0.0
Aspartic acid	g	2.12	1.200
Glutamic acid	g	3.94	3.616
Glycine	g	1.15	0.312
Proline	g	1.28	1.973

Serine	g	1.1	0.888
Vitamin A	IU	1638	400
Vitamin D_3	IU	111	100
Vitamin E	mg	8.9	7.5
Vitamin B_1	mg	1.96	0.6
Vitamin B_2	mg	0.99	0.6
Vitamin B_6	mg	0.87	0.7
Vitamin B_{12}	μg	4.6	2.5
Vitamin K_1	μg	-	75
Vitamin K_3	μg	4	-
Vitamin C	mg	5	-

(In 100g of diet)

Supplementary Table. S2. List of primers used in qPCR.

Gene symbol	Forward primer	Reverse primer
Actb	5'-CATCCGTAAAGACCTCTATGCCAAC-3'	5'-ATGGAGCCACCGATCCACA-3'
Il1b	5'-GCAACTGTTCCTGAACTCAACT-3'	5'-ATCTTTTGGGGTCCGTCAACT-3'
Il6	5'-TCTATACCACTTCACAAGTCGGA-3'	5'-GAATTGCCATTGCACAACTCTTT-3'
Tnf	5'-CCTGTAGCCCACGTCGTAG-3'	5'-GGGAGTAGACAAGGTACAACCC-3'
Tnfrsf1b	5'-TCCTGGCTATTCCCGGAAATG-3'	5'-TGTAAGGATGCTTGGAGTTTGG-3'

Supplementary figure legends:

Supplementary Fig. S1. Experimental design.

Supplementary Fig. S2. Supplementary information about UTUC development in BALB/c female mice. (A) Weekly oral intake of water containing BBN per individual for each strain and sex. (BALB/c female: n = 29, BALB/c male: n = 13, C57BL/6 female: n = 6, C57BL/6 female: n = 5). **(B)** Body weight trend for each BALB/c female individual (n = 19). All BALB/c female mice that lost body weight and were sacrificed developed UTUC.

Supplementary Fig. S3. Supplementary information about the profile of BALB/c female UTUC using multi-omics. (A) Rainfall plots of UN (upper) and UT (lower) showed a single nucleotide variant in each chromosome using WES data (UT: n = 11, UN: n = 5). **(B)** Deconvolution of SBS signatures in UN (upper) and UT (lower), with corresponding COSMIC signatures indicated in parentheses using WES data (UT: n = 11, UN: n = 5). **(C)** Mouse UT mutational plot using the gene list of the OU cohort (UT: n = 11). The gene list contains over 10 % frequent mutation genes in the OU cohort. Red character and red underlined genes are oncogenes. 14/18 genes were mutated in the model's UT sample. **(D)** Gene expression clustering analysis using WTS data showed that UT and U belonged to different clusters. UN seemed to be within U and UT (UT: n = 10, UN: n = 7, U: n = 6).

Supplementary Fig. S4. BALB/c female mice with UTUC mimic human UTUC in multiple aspects. (A) Evaluation of gene expression characteristic of human UTUC molecular subtypes in comparison ⑤ vs ②-④ revealed that FGFR3 markers (left) were downregulated in ⑤. EMT markers (center) and wild-type p53 markers (right) were not significantly different. Mann–Whitney U test was used. *P < 0.05. **(B)** Gene expression of PanCK positive cells was compared between regions of interest (ROI) in ⑤ and ROI in ①, ② (left) and between ROI in ⑤ and ROI in ③, ④ (right) using spatial transcriptome sequencing data. The number of genes upregulated in ⑤ than in ①, ② (left) and ③, ④

(right) were 368 and 132 respectively. The number of genes downregulated in ⑤ than ①, ② (left) and ③, ④ (right) were 467 and 65 respectively. |FC| > 2.0, P value < 0.01 were significant. **(C)** The number of genes distinctively altered in PanCK-positive cells from ROI ⑤.

Supplementary Fig. S5. Elevated inflammatory gene expression in the upper urinary tract is a feature of our model. (A) Gene Ontology (GO) biology process enrichment analysis using WTS data showed the enrichment of GOs related to immune and defense response, epithelial and tissue development, and high positive regulation of gene expression in the UT vs UN (UT: n = 10, UN: n = 7). **(B)** Gene expression of CD3-positive cells was compared between ROI in ⑤ and ③, ④. Four genes were upregulated and none were downregulated (ROI ③: n = 12, ROI ④: n = 3, and ROI ⑤: n = 5). **(C)** Gene expression of F4/80-positive cells was compared between ROI ④,⑤, and ③. 239 genes were upregulated genes and three were downregulated (ROI ③: n = 12, ROI ④: n = 3, and ROI ⑤: n = 5). **(D)** GO enrichment analysis in ④,⑤ vs ③ using F4/80 positive cells showed that ④,⑤ were enriched with GOs involved immune system (ROI ③: n = 12, ROI ④: n = 3, and ROI ⑤: n = 5).

Supplementary Fig. S6. Dietary intervention increases the abundance of *Parabacteroides distasonis* in BALB/c female mice feces. (A and B) α-diversity in gut microbiota were assessed between BALB/c female (Bf, n = 10) vs. others (O, BALB/c male: n = 6, C57BL/6j female: n = 6, and male: n = 5) treated with BBN (A) or BALB/c female treated with BBN (n = 10) vs. tap water (n = 3) (B). Mann–Whitney U test was used. n.s. means not significant. †P < 0.1. **(C and D)** Linear discriminant analysis effect size (LEfSe). Comparison of the abundance of gut microbiota between BALB/c female (n = 10) vs. others (BALB/c male: n = 6, C57BL/6j female: n = 6, and male: n = 5) treated with BBN (C) or BALB/c female treated

with BBN (n = 10) vs. tap water (n = 3) (D). BALB/c female vs. others; Green bar: increased abundance in BALB/c female. Red bar: increased abundance in others. BALB/c female treated BBN vs. Tap water; Green bar: increased abundance in BBN. Red bar: increased abundance in tap water. Microbes underlined in red indicate common microbes in both comparisons. k: kingdom. p: phylum. c: class. o: order. f: family. g: genus. s: species. **(E)** Partial least square-discriminant analysis (PLSDA) of fecal metabolites differentiating Healthy mice from UTUC mice. Red bubble: Healthy. Green bubble: UTUC. **(F)** Correlation analysis between whole fecal metabolites and *genus Parabacteroides* in BALB/c female treated with or without BBN (n = 13). An analysis was performed using the Pearson product-moment correlation coefficient.

Supplementary Fig. S7. Dietary intervention can prevent UTUC carcinogenesis in BALB/c female mice. (A) α-diversity in gut microbiota between the dietary intervention group (n = 8) and normal diet group (n = 8). Mann–Whitney U test was used. $^{*}P < 0.05$. $^{***}P < 0.001$. **(B)** Principal component analysis (PCA) of β-diversity (dietary intervention group: n = 8, normal diet group: n = 8). Red bubble: dietary intervention group. Green bubble: normal diet group. **(C)** Taxa-bar plot of 16S rRNA-seq data in the level of order (o, dietary intervention group: n = 8, normal diet group: n = 8). p: phylum. c: class. o: order. **(D)** Linear discriminant analysis effect size (LEfSe). Red underline bacteria are consistently more or less abundant in BALB/c female mice treated with BBN with UTUC. Green bar (left): increased abundance in the normal diet group. Red bar (right): increased abundance in the dietary intervention group. k: kingdom. p: phylum. c: class. o: order. f: family. g: genus. s: species.

Experimental design

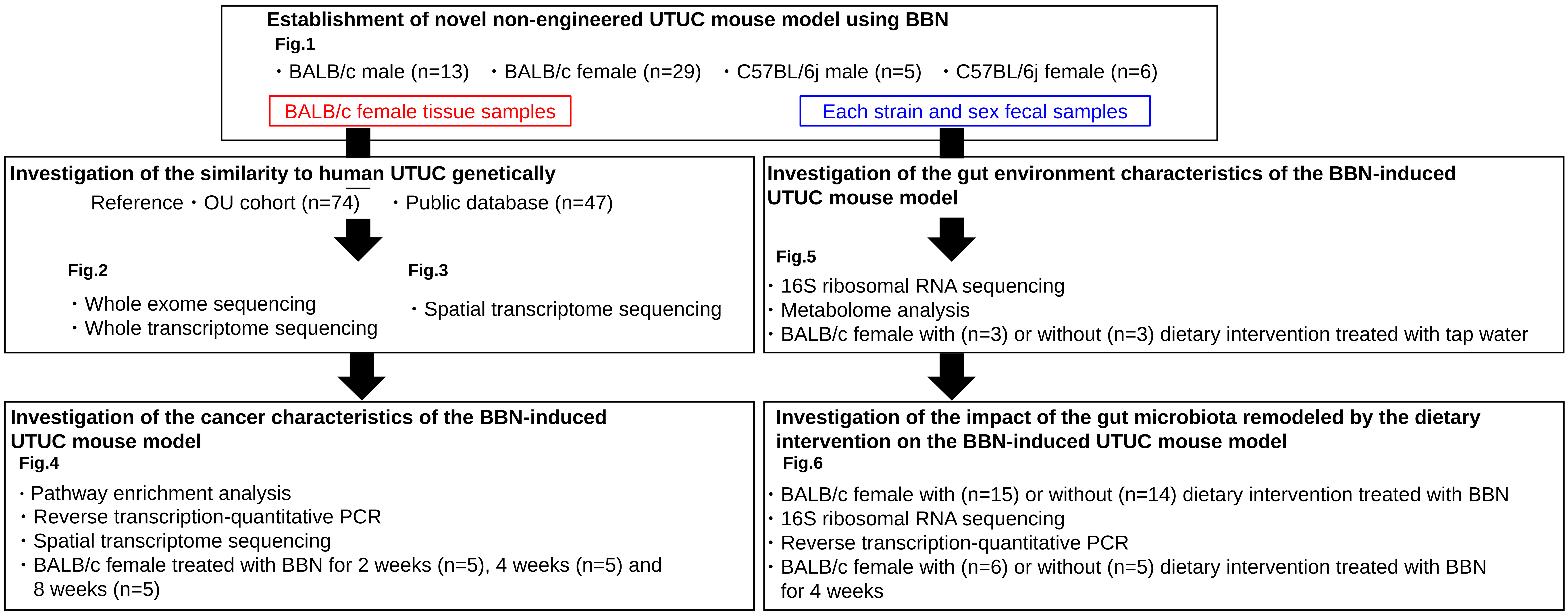

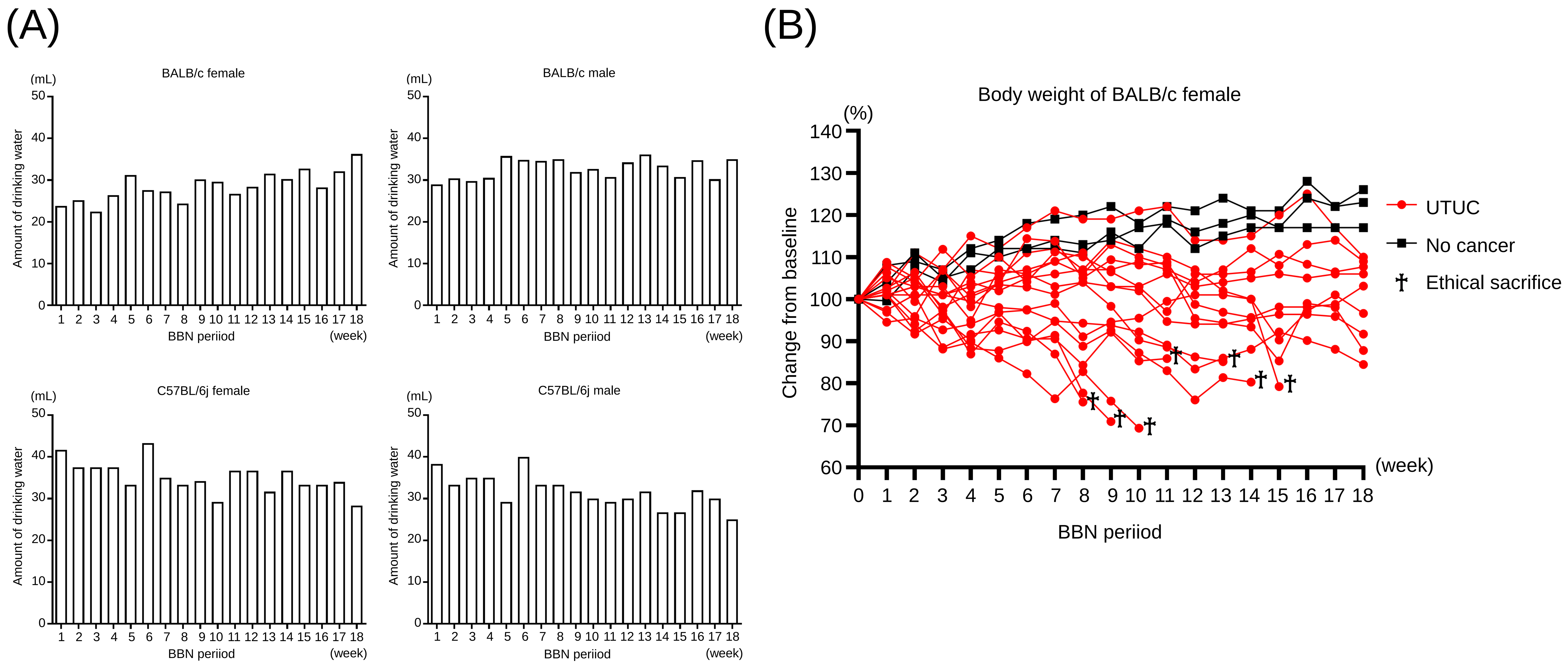
(A)
BALB/c female
BALB/c male
C57BL/6j female
C57BL/6j male
(mL)
Amount of drinking water
BBN periiod
(week)
(B)
Body weight of BALB/c female
(%)
Change from baseline
BBN periiod
(week)
UTUC
No cancer
† Ethical sacrifice

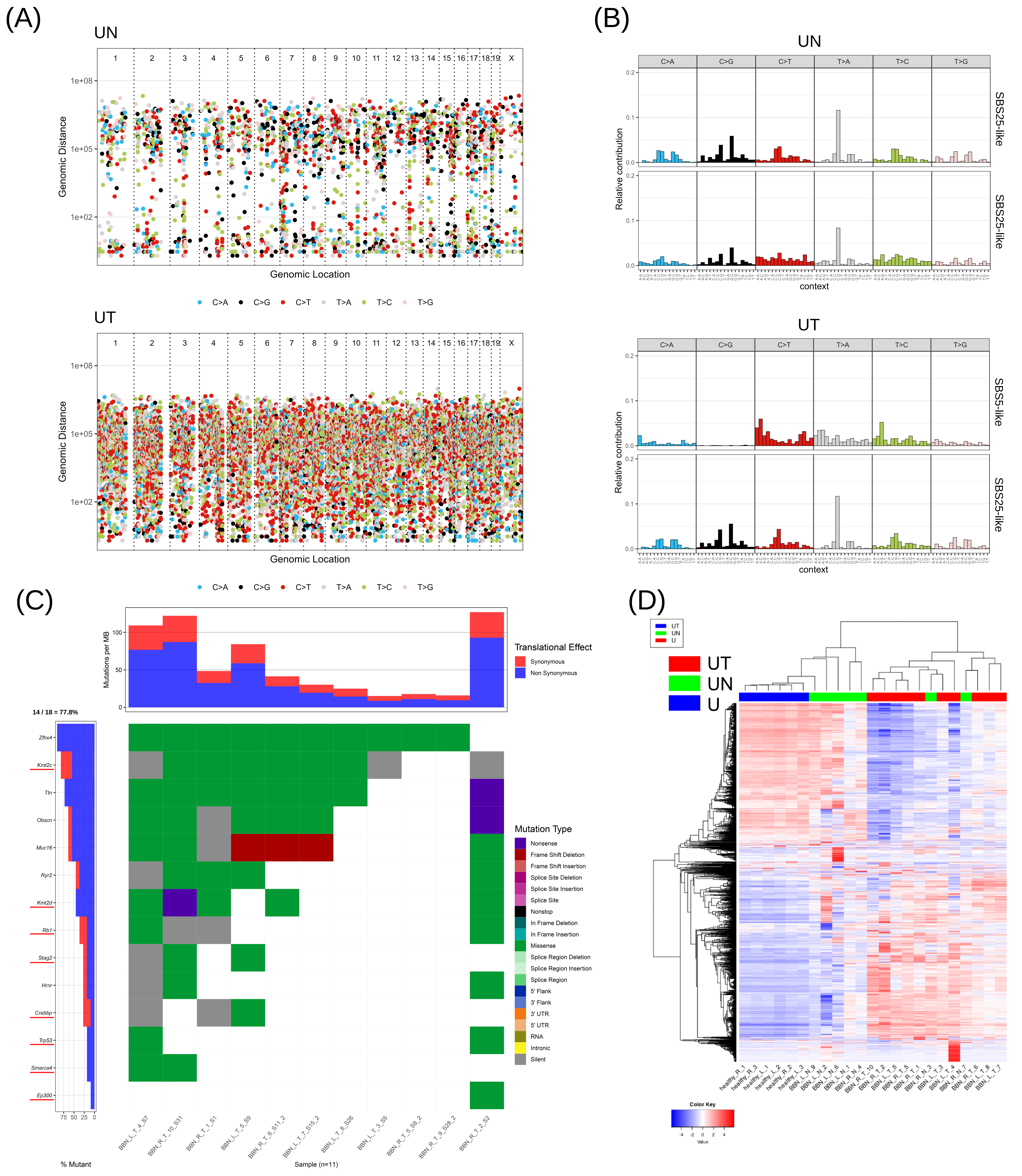
(A)
UN
Genomic Distance
Genomic Location
C>A C>G C>T T>A T>C T>G
UT
(B)
UN
Relative contribution
SBS25-like
context
UT
SBS5-like
(C)
Translational Effect
Synonymous
Non Synonymous
Mutations per MB
14 / 18 = 77.8%
Zfhx4
Kmt2c
Ttn
Obscn
Muc16
Ryr2
Kmt2d
Rb1
Stag2
Hrnr
Crebbp
Trp53
Smarca4
Ep300
% Mutant
Sample (n=11)
Mutation Type
Nonsense
Frame Shift Deletion
Frame Shift Insertion
Splice Site Deletion
Splice Site Insertion
Splice Site
Nonstop
In Frame Deletion
In Frame Insertion
Missense
Splice Region Deletion
Splice Region Insertion
Splice Region
5' Flank
3' Flank
3' UTR
5' UTR
RNA
Intronic
Silent
(D)
UT
UN
U
Color Key
Value

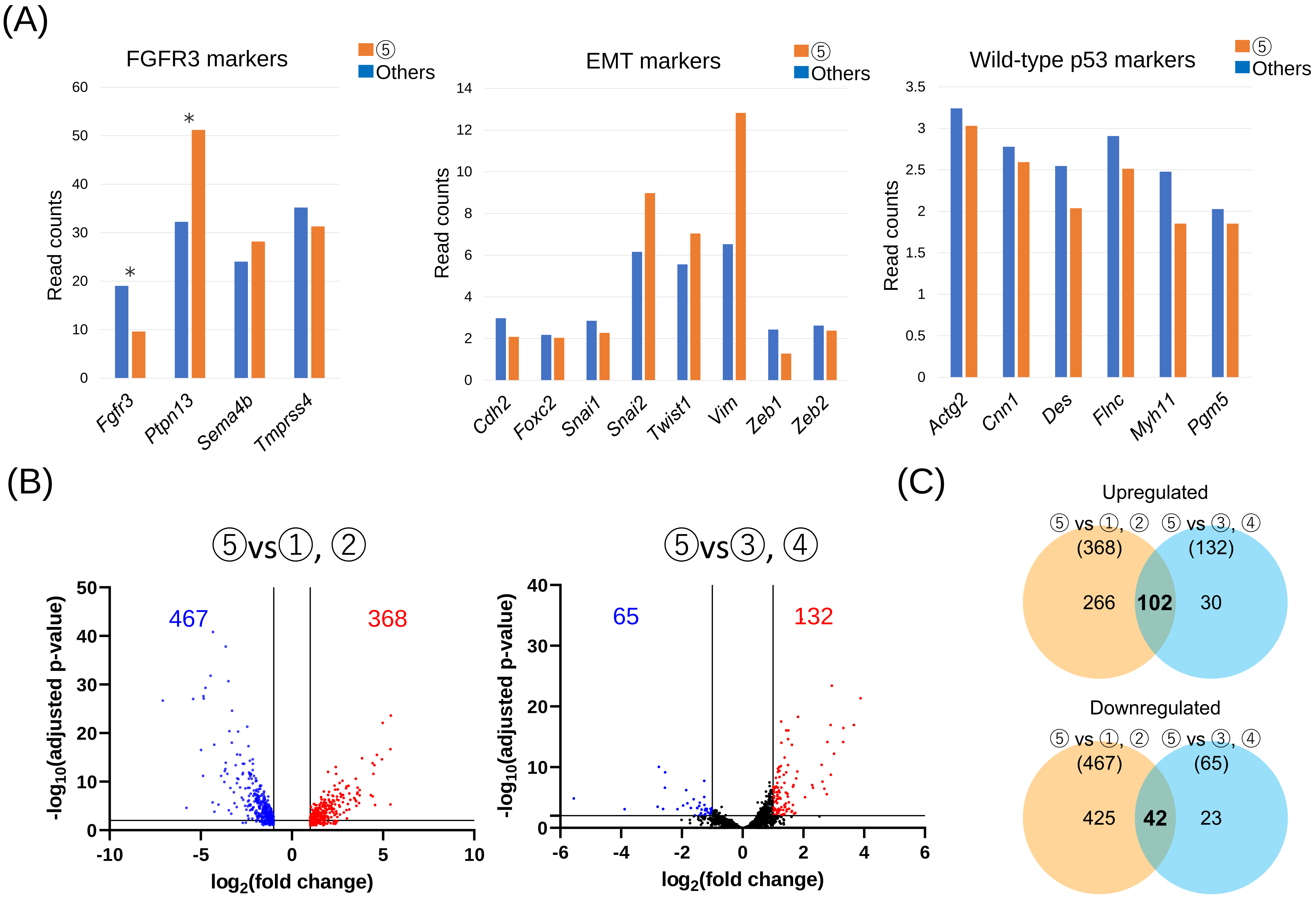
(A)
FGFR3 markers
⑤
Others
Read counts
60
50
40
30
20
10
0
*
*
Fgfr3
Ptpn13
Sema4b
Tmprss4
EMT markers
⑤
Others
Read counts
14
12
10
8
6
4
2
0
Cdh2
Foxc2
Snai1
Snai2
Twist1
Vim
Zeb1
Zeb2
Wild-type p53 markers
⑤
Others
Read counts
3.5
3
2.5
2
1.5
1
0.5
0
Actg2
Cnn1
Des
Flnc
Myh11
Pgm5
(B)
⑤vs①, ②
467
368
-log10(adjusted p-value)
log2(fold change)
50
40
30
20
10
0
-10
-5
0
5
10
⑤vs③, ④
65
132
-log10(adjusted p-value)
log2(fold change)
40
30
20
10
0
-6
-4
-2
0
2
4
6
(C)
Upregulated
⑤ vs ①, ②
(368)
⑤ vs ③, ④
(132)
266
102
30
Downregulated
⑤ vs ①, ②
(467)
⑤ vs ③, ④
(65)
425
42
23

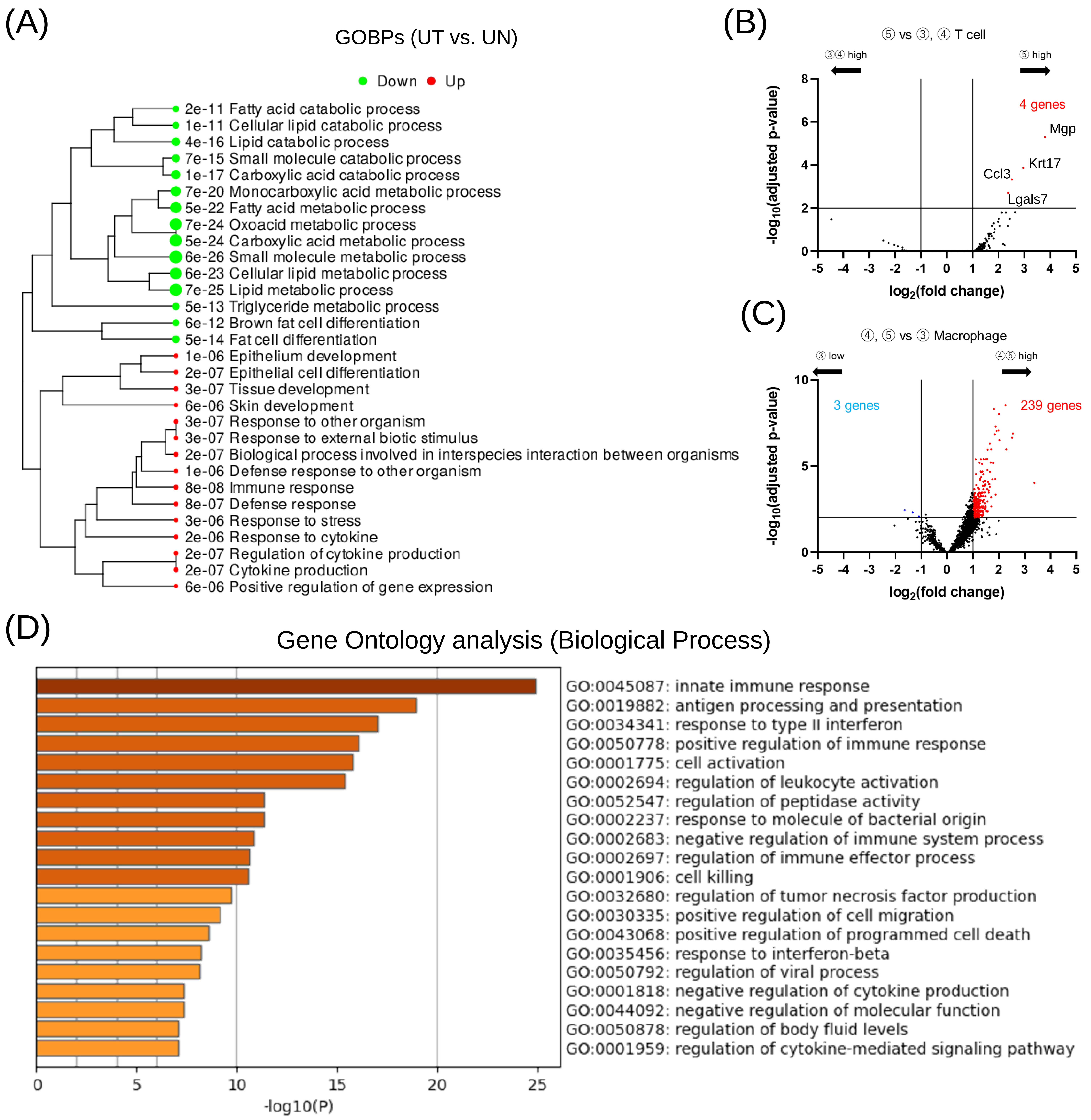
(A)
GOBPs (UT vs. UN)
Down Up
2e-11 Fatty acid catabolic process
1e-11 Cellular lipid catabolic process
4e-16 Lipid catabolic process
7e-15 Small molecule catabolic process
1e-17 Carboxylic acid catabolic process
7e-20 Monocarboxylic acid metabolic process
5e-22 Fatty acid metabolic process
7e-24 Oxoacid metabolic process
5e-24 Carboxylic acid metabolic process
6e-26 Small molecule metabolic process
6e-23 Cellular lipid metabolic process
7e-25 Lipid metabolic process
5e-13 Triglyceride metabolic process
6e-12 Brown fat cell differentiation
5e-14 Fat cell differentiation
1e-06 Epithelium development
2e-07 Epithelial cell differentiation
3e-07 Tissue development
6e-06 Skin development
3e-07 Response to other organism
3e-07 Response to external biotic stimulus
2e-07 Biological process involved in interspecies interaction between organisms
1e-06 Defense response to other organism
8e-08 Immune response
8e-07 Defense response
3e-06 Response to stress
2e-06 Response to cytokine
2e-07 Regulation of cytokine production
2e-07 Cytokine production
6e-06 Positive regulation of gene expression
(B)
⑤ vs ③, ④ T cell
③④ high
⑤ high
4 genes
Mgp
Krt17
Ccl3
Lgals7
-log10(adjusted p-value)
log2(fold change)
(C)
④, ⑤ vs ③ Macrophage
③ low
④⑤ high
3 genes
239 genes
-log10(adjusted p-value)
log2(fold change)
(D)
Gene Ontology analysis (Biological Process)
GO:0045087: innate immune response
GO:0019882: antigen processing and presentation
GO:0034341: response to type II interferon
GO:0050778: positive regulation of immune response
GO:0001775: cell activation
GO:0002694: regulation of leukocyte activation
GO:0052547: regulation of peptidase activity
GO:0002237: response to molecule of bacterial origin
GO:0002683: negative regulation of immune system process
GO:0002697: regulation of immune effector process
GO:0001906: cell killing
GO:0032680: regulation of tumor necrosis factor production
GO:0030335: positive regulation of cell migration
GO:0043068: positive regulation of programmed cell death
GO:0035456: response to interferon-beta
GO:0050792: regulation of viral process
GO:0001818: negative regulation of cytokine production
GO:0044092: negative regulation of molecular function
GO:0050878: regulation of body fluid levels
GO:0001959: regulation of cytokine-mediated signaling pathway
-log10(P)

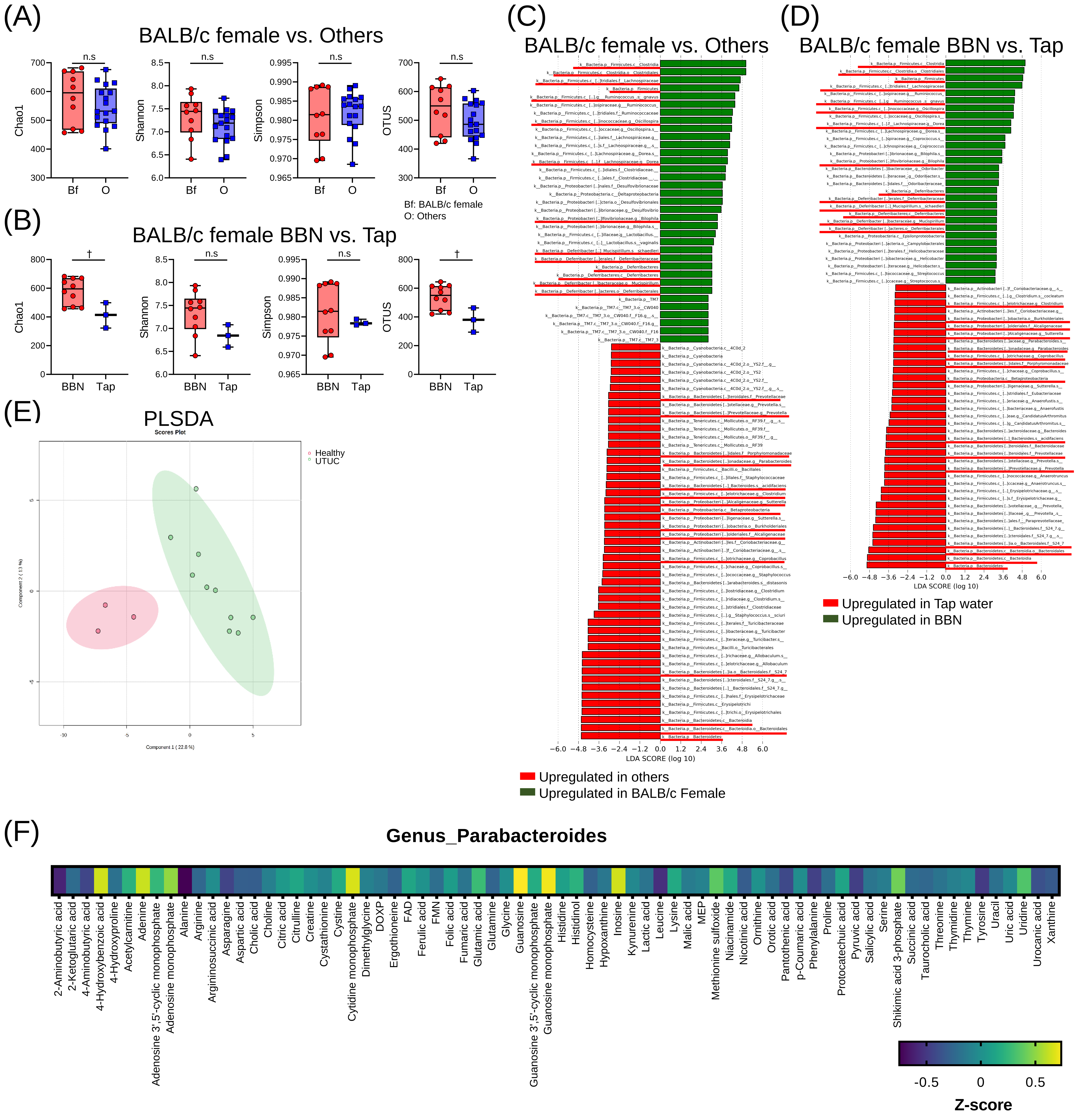
(A)
BALB/c female vs. Others
Chao1
Shannon
Simpson
OTUS
n.s
Bf
O
Bf: BALB/c female
O: Others
(B)
BALB/c female BBN vs. Tap
†
BBN
Tap
(C)
BALB/c female vs. Others
LDA SCORE (log 10)
Upregulated in others
Upregulated in BALB/c Female
(D)
BALB/c female BBN vs. Tap
LDA SCORE (log 10)
Upregulated in Tap water
Upregulated in BBN
(E)
PLSDA
Scores Plot
Healthy
UTUC
Component 1 (22.8 %)
Component 2 (13 %)
(F)
Genus_Parabacteroides
2-Aminobutyric acid
2-Ketoglutaric acid
4-Aminobutyric acid
4-Hydroxybenzoic acid
4-Hydroxyproline
Acetylcarnitine
Adenine
Adenosine 3',5'-cyclic monophosphate
Adenosine monophosphate
Alanine
Arginine
Argininosuccinic acid
Asparagine
Aspartic acid
Cholic acid
Choline
Citric acid
Citrulline
Creatine
Cystathionine
Cystine
Cytidine monophosphate
Dimethylglycine
DOXP
Ergothioneine
FAD
Ferulic acid
FMN
Folic acid
Fumaric acid
Glutamic acid
Glutamine
Glycine
Guanosine
Guanosine 3',5'-cyclic monophosphate
Guanosine monophosphate
Histidine
Histidinol
Homocysteine
Hypoxanthine
Inosine
Kynurenine
Lactic acid
Leucine
Lysine
Malic acid
MEP
Methionine sulfoxide
Niacinamide
Nicotinic acid
Ornithine
Orotic acid
Pantothenic acid
p-Coumaric acid
Phenylalanine
Proline
Protocatechuic acid
Pyruvic acid
Salicylic acid
Serine
Shikimic acid 3-phosphate
Succinic acid
Taurocholic acid
Threonine
Thymidine
Tyrosine
Uracil
Uric acid
Uridine
Urocanic acid
Xanthine
-0.5
0
0.5
Z-score

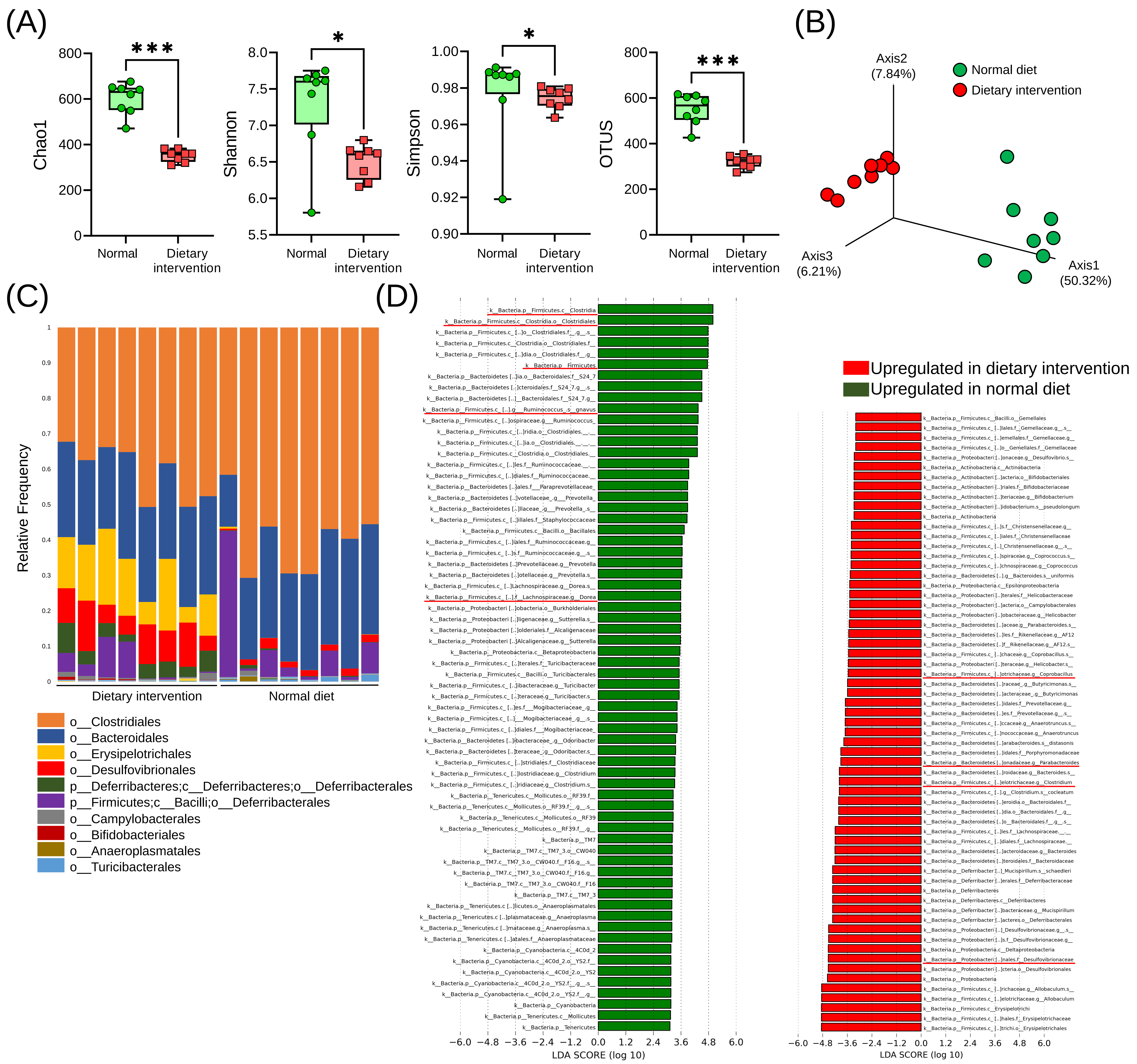
(A)
Chao1
Shannon
Simpson
OTUS
Normal
Dietary intervention
***
*
(B)
Axis1 (50.32%)
Axis2 (7.84%)
Axis3 (6.21%)
Normal diet
Dietary intervention
(C)
Relative Frequency
Dietary intervention
Normal diet
o__Clostridiales
o__Bacteroidales
o__Erysipelotrichales
o__Desulfovibrionales
p__Deferribacteres;c__Deferribacteres;o__Deferribacterales
p__Firmicutes;c__Bacilli;o__Deferribacterales
o__Campylobacterales
o__Bifidobacteriales
o__Anaeroplasmatales
o__Turicibacterales
(D)
Upregulated in dietary intervention
Upregulated in normal diet
LDA SCORE (log 10)
